# Integrating Fluorescence from Self-Trapped Excitons and Phosphorescence in Zero-Dimensional Metal Halides for Time-Resolved Dynamic Information Encryption

**DOI:** 10.3390/nano16171121

**Published:** 2026-09-07

**Authors:** Xiang Zhu, Lei Li, Fei Wen, Yu Wang, Yangbin Xu, Zhixuan Wang, Cuixia You, Qingchun Chen, Lingling Xu, Jiansong Ye, Jiaxing Song, Nengchao Qiu, Yanxing Feng, Tingwei He, Hai Jia, Quanlin Chen

**Affiliations:** 1 Key Laboratory of Featured Materials in Biochemical Industry, College of New Energy and Materials, Ningde Normal University, Ningde 352100, China; isolate315@gmail.com (X.Z.); 15336353081@163.com (L.L.); 18314812570@163.com (F.W.); 15064424068@163.com (Y.W.); 18750517889@163.com (Y.X.); 18059370355@163.com (Z.W.); 15260895831@139.com (C.Y.); 18259386955@163.com (Q.C.); 17775732846@163.com (L.X.); 15160575260@163.com (J.Y.); 18960886633@163.com (J.S.); 13620040418@163.com (N.Q.); 2 Institute of Advanced Ceramics, Henan Academy of Sciences, Zhengzhou 450046, China; fengyanxing@stu.htu.edu.cn; 3 Hebei Key Laboratory of Optic-Electronic Information and Materials, Province-Ministry Co-Construction Collaborative Innovation Center of Hebei Photovoltaic Technology, College of Physics Science and Technology, Hebei University, Baoding 071002, China; 4 College of Mathematics and Physics, Ningde Normal University, Ningde 352100, China

**Keywords:** 0D metal halides, room-temperature phosphorescence, localized/self-trapped excitonic emission, energy transfer, time-gated optical information encoding

## Abstract

Multimodal luminescent materials integrating spectral and temporal information are highly desirable for dynamic optical information encoding. However, constructing such systems often requires complicated molecular design or multiple synthetic steps. Herein, we report a simple Sb-introduction strategy to regulate excited-state dynamics in the zero-dimensional (0D) organic–inorganic hybrid metal halide (AP)_2_ZnCl_4_ (AP = 2-aminoacetophenone). The pristine host intrinsically combines prompt AP^+^ fluorescence with long-lived AP^+^-derived room-temperature phosphorescence (RTP). Upon Sb introduction, an additional broad Sb-related localized/self-trapped excitonic emission appears and the excited-state relaxation kinetics are redistributed while the native RTP pathway remains operative. These composition-dependent responses enable a proof-of-concept sequential time-gated optical encoding/decoding scheme with “WWW”, “SUV”, and “RTP” outputs. The results highlight dopant-mediated excited-state regulation in 0D hybrid metal halides for dynamic optical information encoding.

## 1. Introduction

Optical materials capable of room-temperature phosphorescence (RTP) and spectrally resolvable emission have attracted sustained interest for information encryption and time-dependent data storage. Compared with single-mode emitters, systems that integrate emission wavelength, intensity, and lifetime can encode information across multiple optical dimensions, enabling more complex anti-counterfeiting schemes [1,2,3,4,5]. Currently, organic RTP materials have been extensively investigated due to their structural tunability and solution processability. However, achieving additional emission modalities in purely organic systems generally relies on elaborate molecular engineering or multistep synthetic modifications, limiting practical scalability [1,6,7,8]. Developing simpler compositional strategies to regulate multi-modal luminescence therefore remains a key challenge.

Low-dimensional organic–inorganic metal halides, particularly 0D architectures, have emerged as promising platforms for manipulating excited-state processes. In these materials, isolated metal-halide polyhedra are spatially separated by organic ionic matrices, resulting in strong quantum confinement and localized excitonic states. Such unique structural features enable flexible regulation of photophysical properties through compositional engineering, including metal-site substitution, dopant incorporation, and defect modulation [9,10,11,12]. Among these strategies, introducing optically active dopants has proven to be an effective approach for generating additional emissive centers and tailoring excited-state dynamics. Recent studies have demonstrated that dopant incorporation can induce new luminescent pathways, enabling dual emission, tunable afterglow, and enhanced information storage capability [13,14,15]. Nevertheless, the coexistence of multiple emissive centers requires precise control over excited-state interactions, as uncontrolled energy transfer or competitive relaxation processes may influence emission efficiency and lifetime characteristics [16].

To improve emission efficiency while maintaining spectral versatility, dopants with ns^2^ electronic configurations (e.g., Sb^3+^, Bi^3+^, Te^4+^) have emerged as promising candidates. Their lone-pair s electrons can couple strongly with host band-edge states, promoting electronic localization, lattice relaxation, and broad-band radiative recombination. Such localized/self-trapped excitonic emission typically exhibits a broad profile and large Stokes shift [14,17,18,19,20,21,22,23]. Moreover, introducing ns^2^ dopants into organic–inorganic hybrid hosts offers the possibility of integrating inorganic localized emission with organic phosphorescence, thereby creating complementary fluorescence/phosphorescence channels within a single material. However, the excited-state coupling between dopant-induced inorganic emission centers and organic triplet-related RTP processes remains insufficiently understood. In particular, achieving energy redistribution while preserving long-lived organic afterglow represents a critical challenge for designing multifunctional hybrid luminescent materials [13,14].

Herein, we develop a multi-channel excited-state regulation strategy using the 0D organic–inorganic hybrid compound (AP)_2_ZnCl_4_ (AP = 2-aminoacetophenone; AP^+^ = the protonated form of AP). A key distinction of this system is that the pristine host already possesses two emissive channels with markedly different temporal characteristics, namely prompt AP^+^ fluorescence and long-lived AP^+^-derived RTP. Upon Sb introduction, spectroscopic and time-resolved measurements support an additional Sb-related localized emissive channel and an extra relaxation pathway from the organic singlet manifold while the native RTP pathway remains operative. The resulting composition-dependent temporal responses are converted into sequential time-gated optical readout within a single material family. This work therefore emphasizes dopant-regulated redistribution among pre-existing prompt and triplet channels together with an added Sb-related localized channel, rather than dopant-induced broadband emission alone, and demonstrates a proof-of-concept strategy for dynamic optical information encoding.

## 2. Materials and Methods

### 2.1. Materials

Antimony trichloride (SbCl_3_, analytical reagent grade), 2-aminoacetophenone (C_8_H_9_NO, analytical reagent grade), zinc chloride (ZnCl_2_, analytical reagent grade), concentrated hydrochloric acid (HCl, analytical reagent grade), and diisopropyl ether (C_6_H_14_O, analytical reagent grade) were purchased from Shanghai Macklin Biochemical Co., Ltd. (Shanghai, China). All reagents were used as received without further purification.

### 2.2. Crystal Growth of (AP)_2_ZnCl_4_·H_2_O

ZnCl_2_ (2.00 mmol, 272.6 mg) and AP (800 μL) were mixed with 4.00 mL of concentrated hydrochloric acid in a 10 mL glass vial. The mixture was heated at 100 °C under constant magnetic stirring for 20 min until a clear solution was obtained. The resulting solution was allowed to cool to room temperature over approximately 30 min and was subsequently kept undisturbed at room temperature for 48 h to promote crystal growth. The resulting crystals were collected by filtration, washed three times with diisopropyl ether, and dried in a forced-air oven at 60 °C for 5 min. The dried crystals were stored in sealed sample bags for subsequent characterization.

### 2.3. Crystal Growth of Nominally Sb-Modified (AP)_2_ZnCl_4_·H_2_O

Nominally Sb-modified crystals, denoted as (AP)_2_ZnCl_4_:*x*%Sb (*x* = 1, 3, 5, 7, 10, and 15), were prepared using a slow-cooling crystallization method. Here, *x* represents the nominal molar percentage of Sb relative to the total amount of Zn and Sb in the precursor solution: *x* = *n*_Sb_/(*n*_Zn_ + *n*_Sb_) × 100%.

For each synthesis, the total amount of ZnCl_2_ and SbCl_3_ was maintained at 2.00 mmol. The amounts of ZnCl_2_ used for *x* = 1, 3, 5, 7, 10, and 15% were 1.98, 1.94, 1.90, 1.86, 1.80, and 1.70 mmol, respectively. A 0.50 M SbCl_3_ stock solution was freshly prepared by dissolving SbCl_3_ (2.50 mmol) in concentrated hydrochloric acid. The required volumes of SbCl_3_ stock solution (40, 120, 200, 280, 400, and 600 μL) were added to obtain the samples with different Sb concentrations. The subsequent crystal growth process was identical to that of pristine (AP)_2_ZnCl_4_·H_2_O.

### 2.4. Crystal Growth of 2-Aminoacetophenone Hydrochloride (APCl)

APCl crystals were prepared using a procedure similar to that employed for the hybrid crystals. Briefly, AP (800 μL) was mixed with 4.00 mL of concentrated hydrochloric acid in a 10 mL glass vial. The mixture was heated at 100 °C under constant magnetic stirring for 20 min until a clear solution was obtained. The resulting solution was allowed to cool to room temperature over approximately 30 min and was subsequently kept undisturbed at room temperature for 48 h to promote crystal growth. The resulting APCl crystals were collected by filtration, washed three times with diisopropyl ether, and dried in a forced-air oven at 60 °C for 5 min. The dried crystals were stored in sealed sample bags for subsequent characterization.

### 2.5. Characterization

Crystal-packing representations of APCl (Table S1) and the parent (AP)_2_ZnCl_4_·H_2_O (Table S2) phase were generated from previously reported crystallographic information files (CCDC 2321893 and 2321895, respectively) using VESTA (version 3.5.7). Powder X-ray diffraction (PXRD) patterns were recorded at room temperature on a D8 Advance X-ray diffractometer (Bruker AXS GmbH, Karlsruhe, Germany) using Cu Kα radiation (*λ* = 1.5418 Å), operated at 40 kV and 40 mA. Data were collected over 2*θ* = 5–60° with a step size of 0.0215° and a counting time of 0.1 s per step. Ultraviolet–visible diffuse-reflectance spectra were measured using a UV-2600 UV–Vis spectrophotometer equipped with an ISR-2600Plus integrating-sphere attachment (Shimadzu Corporation, Kyoto, Japan), with the instrument-supplied BaSO_4_ reference used as the 100% reflectance standard, and the reflectance data were transformed using the Kubelka–Munk function. Steady-state photoluminescence (PL), photoluminescence excitation (PLE), phosphorescence-mode spectra, and excitation–emission matrices were recorded at room temperature using an F-4700 fluorescence spectrophotometer (Hitachi High-Tech Corporation, Tokyo, Japan) with 5.0 nm excitation and emission slit widths; phosphorescence-mode measurements used a 40 Hz chopping frequency. Each point in the excitation–emission matrices corresponds to an experimentally acquired measurement point. No post-acquisition smoothing, resampling, interpolation to additional data points, or merging of neighboring measurements was applied; the apparent continuity in the contour maps arises only from contour rendering. Absolute photoluminescence quantum yields were measured using a 60 mm Integrating Sphere Unit (Part No. 250-0123; Hitachi High-Tech Corporation, Tokyo, Japan) coupled to the F-4700 fluorescence spectrophotometer. Time-resolved PL decay profiles were collected under 360 nm pulsed excitation using an FLS1000 photoluminescence spectrometer (Edinburgh Instruments Ltd., Livingston, UK) and monitored at 430 nm for the nanosecond-scale donor fluorescence and at 620 nm for the microsecond-scale Sb-related emission. The decay traces were analyzed using Fluoracle software (version 1.4.2) and fitted using a biexponential model, *I*(*t*) = *I*_0_ + *A*_1_exp(−*t*/*τ*_1_) + *A*_2_exp(−*t*/*τ*_2_), and the reported average lifetime was calculated using the intensity-weighted expression *τ_avg_* = (*A*_1_*τ*_1_^2^ + *A*_2_*τ*_2_^2^)/(*A*_1_*τ*_1_ + *A*_2_*τ*_2_). Time-resolved phosphorescence decay profiles monitored at 525 nm were recorded using the F-4700 fluorescence spectrophotometer and fitted using a biexponential decay model in OriginPro 2024 (OriginLab Corporation, Northampton, MA, USA). Power-dependent PL spectra were recorded under 360 nm continuous-wave excitation using an MDL-III-360 laser (Changchun New Industries Optoelectronics Tech. Co., Ltd., Changchun, China). The excitation power density was varied from 100 to 400 mW cm^−2^ using neutral-density filters; the incident power was monitored using a PM100D optical power meter equipped with an S120VC silicon photodiode sensor (Thorlabs Inc., Newton, NJ, USA). Time-gated afterglow photographs were recorded under 365 nm excitation using a ZF-7A ultraviolet lamp (Shanghai Guanghao Analytical Instrument Co., Ltd., Shanghai, China). SEM–EDS elemental mapping of the nominal 5% Sb sample was performed using a GeminiSEM 300 scanning electron microscope (Carl Zeiss Microscopy GmbH, Oberkochen, Germany) equipped with an X-Max 50 silicon-drift EDS detector (Oxford Instruments NanoAnalysis, High Wycombe, UK) to examine the elemental distribution. EDS data were acquired and analyzed using AZtec software (version 5.0). A representative repeatability analysis of the delayed PL spectra of pristine (AP)_2_ZnCl_4_ under matched optical settings is provided in Figure S12.

### 2.6. Computational Methods

Density functional theory (DFT) calculations were performed using the Vienna Ab Initio Simulation Package (VASP, version 5.4.4) with the projector-augmented-wave (PAW) method. Structural relaxation and electronic-structure calculations were carried out within the generalized gradient approximation using the Perdew–Burke–Ernzerhof (GGA-PBE) exchange-correlation functional. A plane-wave cutoff energy of 350 eV was employed. The electronic convergence criterion was set to 1 × 10^−2^ eV, and ionic relaxation was continued until the residual force on each atom was below 0.05 eV Å^−1^. The resulting DOS/PDOS calculations are used as supportive ground-state electronic-structure context and are not treated as standalone proof of a unique Sb site, Förster-type energy transfer, or excited-state self-trapping.

## 3. Results and Discussion

### 3.1. Materials Characterization

**Figure 1:**
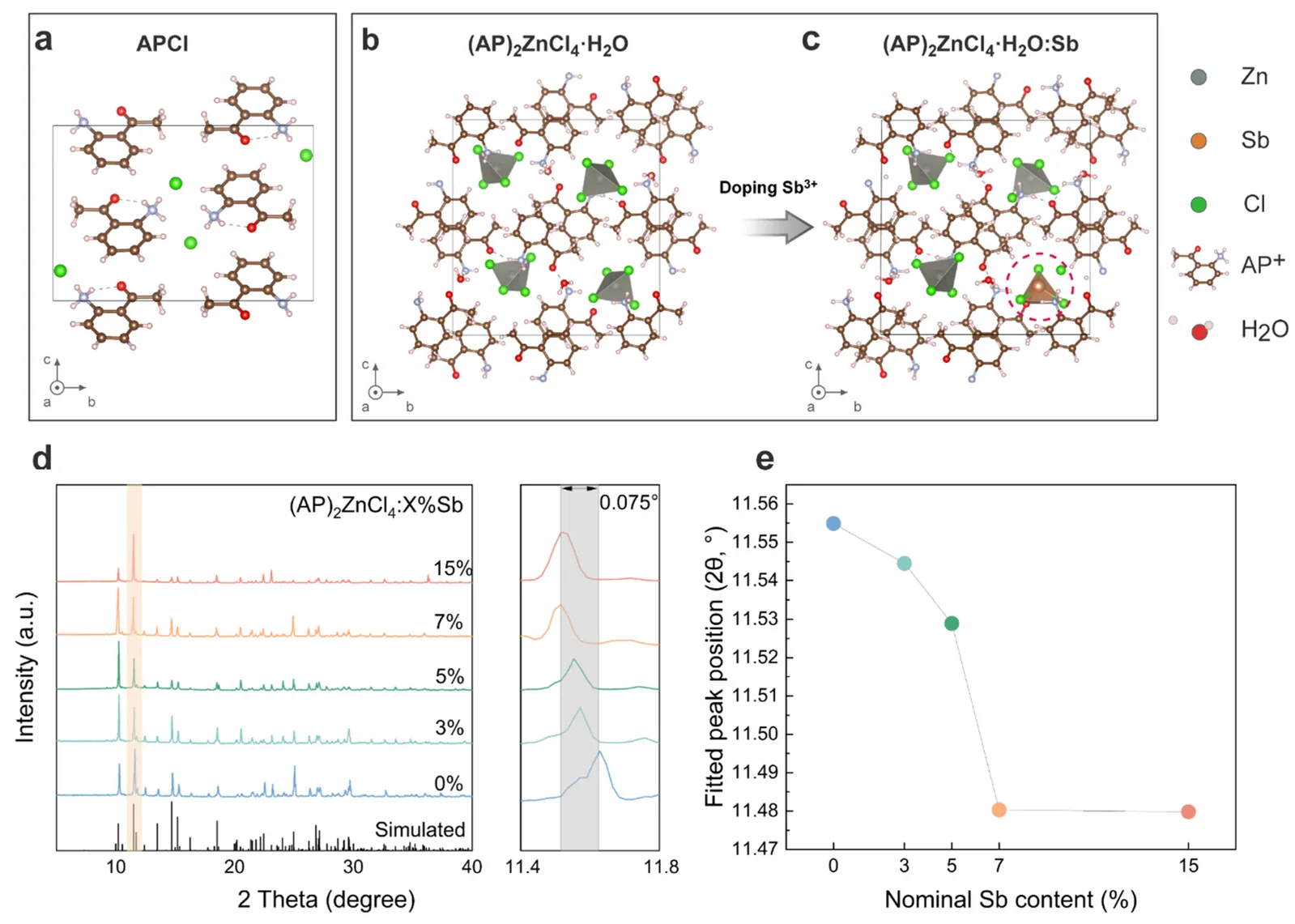
Structural characteristics of pristine and nominally Sb-modified (AP)_2_ZnCl_4_. (**a**–**c**) Crystal-packing schematics of APCl, pristine (AP)_2_ZnCl_4_, and a schematic possible Sb-associated local environment, respectively. (**d**) PXRD patterns of (AP)_2_ZnCl_4_:*x*%Sb (*x* = 0, 3, 5, 7, and 15) together with an enlarged view of the selected low-angle reflection. (**e**) Fitted peak position of the selected reflection as a function of nominal Sb content. The letters a, b, and c in the structural schematics denote the crystallographic axes.

We selected the AP^+^ cation as the organic component. The coexistence of carbonyl oxygen and amino nitrogen introduces lone-pair electrons, allowing both conventional *π* → π* and *n* → π* electronic transitions (Figure 1a). The participation of *n* → π* excited states facilitates intersystem crossing (ISC) according to El-Sayed’s rule, making AP^+^ a suitable building block for RTP [24,25]. ZnCl_2_ was employed as the inorganic precursor because the chloride ligands provide a moderate heavy-atom effect that enhances spin–orbit coupling (SOC), while the closed-shell *d*^10^ electronic configuration of Zn^2+^ minimizes competing *d*–*d* transitions and effectively preserves the excited-state energy of the organic chromophore. The combination of these features is expected to promote efficient triplet-state generation while suppressing non-radiative energy dissipation [24,26].

The organic–inorganic hybrid metal halide (AP)_2_ZnCl_4_·H_2_O (hereafter denoted as (AP)_2_ZnCl_4_) was synthesized by a slow-cooling crystallization method using AP as the organic ligand. According to the previously reported crystallographic data (Table S2) [24], the parent phase adopts a monoclinic 0D structure in which isolated [ZnCl_4_]^2−^ tetrahedra are completely separated by AP^+^ cations (Figure 1b). The inorganic tetrahedra are stabilized primarily through electrostatic interactions with the surrounding organic cations, resulting in strong spatial confinement that suppresses long-range electronic coupling and favors exciton localization. Lattice water molecules further act as structural bridges, constructing an extended supramolecular network through N–H⋯Cl, C–H⋯Cl, N–H⋯O, and O–H⋯O hydrogen bonds together with *π*–*π* stacking interactions. By comparison, APCl is governed predominantly by organic molecular packing and lacks the same extended organic–inorganic supramolecular network (Figure 1a; Table S1). This structural contrast indicates that the hybrid lattice provides a more rigid environment for AP^+^ cations, suppressing molecular vibrations and rotations and thereby reducing non-radiative decay pathways that compete with long-lived RTP emission [24].

Nominally Sb-modified crystals, denoted as (AP)_2_ZnCl_4_:*x*%Sb, were subsequently prepared using the same crystallization procedure. Figure 1c is presented only as a schematic representation of a possible Sb-associated local environment and is not intended to establish site-specific substitution. The phase purity and average lattice structure were examined by PXRD. As shown in Figure 1d, the diffraction profiles of all compositions remain consistent with the parent (AP)_2_ZnCl_4_ phase, with no additional reflections detected within the measurement range. The selected low-angle reflection shifts from approximately 11.555° for *x* = 0 to approximately 11.480° for *x* = 7 and remains nearly unchanged at *x* = 15. The corresponding fitted peak-position trend is summarized in Figure 1e, giving an overall low-angle shift of about 0.075°. We therefore interpret the PXRD evolution conservatively as a composition-dependent perturbation of the average lattice environment rather than direct proof of a specific Zn^2+^/Sb^3+^ substitutional site. Such an average-lattice perturbation is chemically plausible given the different effective ionic radii of Zn^2+^ and Sb^3+^ [27], although the present data do not establish substitution. Complementary SEM–EDS elemental mapping of the nominal 5% Sb sample (Figure S1) shows that the Sb signal is distributed across the analyzed region without obvious large-scale Sb-rich segregation. These data support the presence and micrometer-scale distribution of Sb, but do not determine its crystallographic site or local coordination environment.

**Figure 2:**
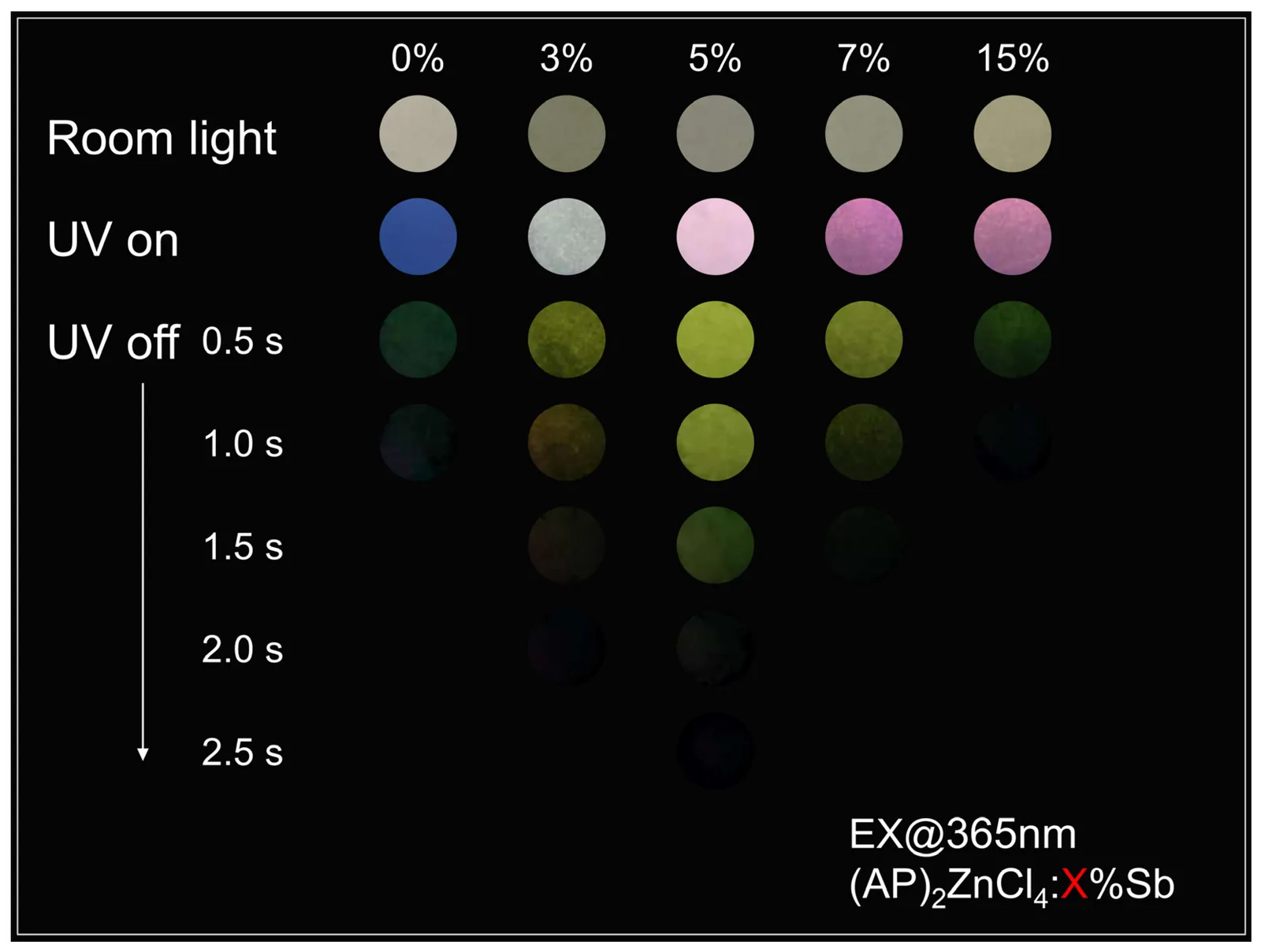
Optical appearances and afterglow evolution of pristine and nominally Sb-modified (AP)_2_ZnCl_4_:*x*%Sb (*x* = 0, 3, 5, 7, and 15). Photographs were recorded under room light, under 365 nm ultraviolet irradiation, and at 0.5, 1.0, 1.5, 2.0, and 2.5 s after removal of the excitation source.

The optical response of the resulting crystals was then investigated. Under room light, the pristine and nominally Sb-modified samples show similar pale appearances (Figure 2). Under 365 nm UV irradiation, however, the prompt-emission color evolves markedly with nominal Sb content, changing from blue for pristine (AP)_2_ZnCl_4_ to pink for the Sb-containing compositions. After the excitation source is removed, a green afterglow remains for all samples; for selected compositions, visible emission persists to approximately 2.0–2.5 s. These observations show that Sb introduction regulates the prompt-emission color while the intrinsic RTP remains operative.

### 3.2. Prompt and Delayed Photoluminescence Properties

**Figure 3:**
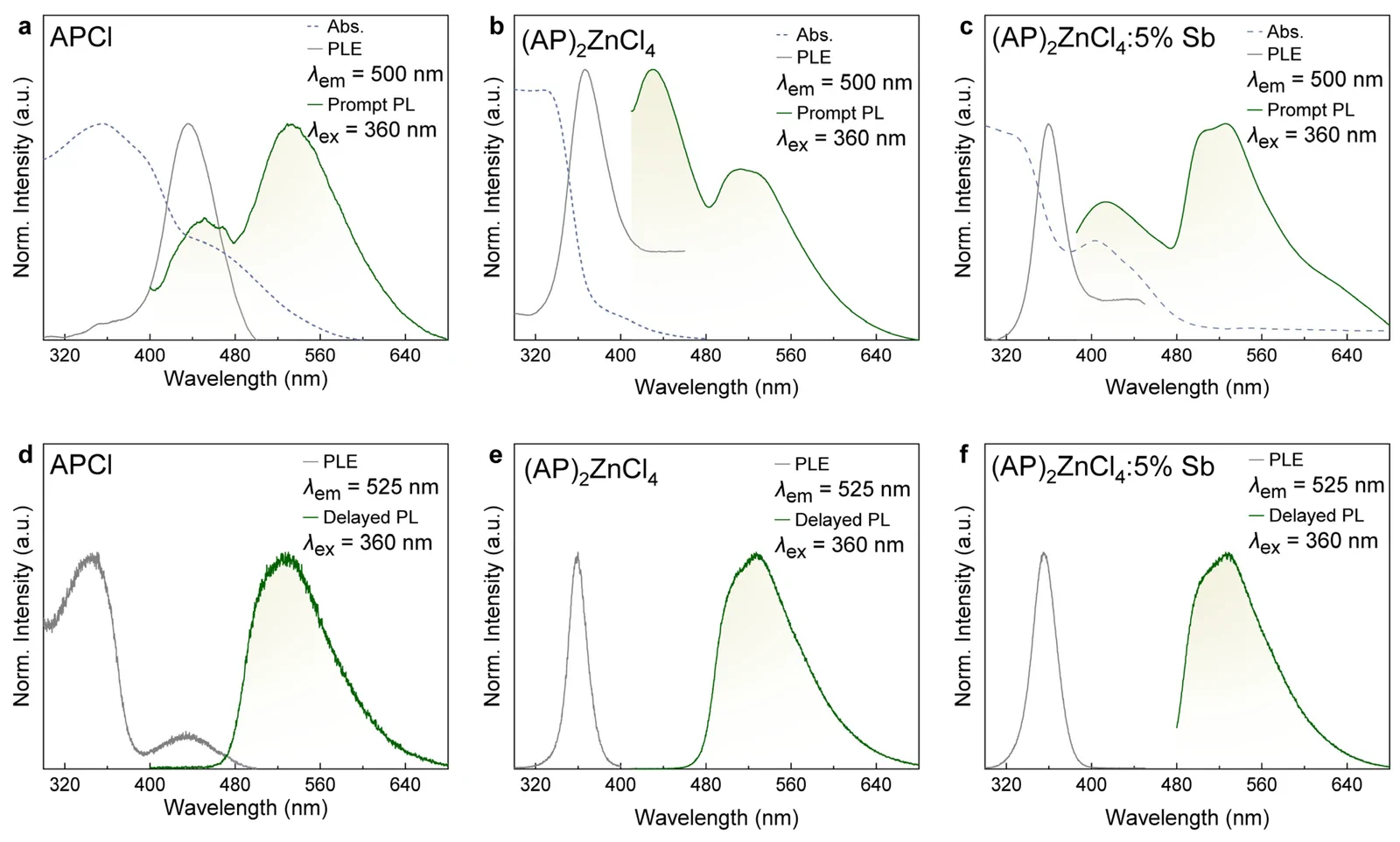
Steady-state and delayed photoluminescence properties of APCl, (AP)_2_ZnCl_4_, and (AP)_2_ZnCl_4_:5%Sb. (**a**–**c**) Ultraviolet–visible absorption spectra (blue dashed lines), PLE spectra monitored at 500 nm (gray lines), and prompt PL spectra recorded under 360 nm excitation (green lines). (**d**–**f**) PLE spectra monitored at 525 nm (gray lines) and delayed PL spectra recorded under 360 nm excitation (green lines) for the corresponding samples.

To elucidate the origin of the emission-color evolution in (AP)_2_ZnCl_4_:*x*%Sb, the prompt PL properties were systematically investigated under 360 nm excitation. As shown in Figure 3a,b (green curves), (AP)_2_ZnCl_4_ exhibits two emission bands centered at 430 and 500 nm, closely resembling the intrinsic emission profile of APCl, suggesting that the luminescence is primarily derived from the organic AP^+^ chromophore. In the delayed PL spectra of APCl and (AP)_2_ZnCl_4_ (Figure 3d,e; Figure S2), the short-wavelength emission at 430 nm disappears completely, whereas the long-lived emission (500–530 nm) region remains. This indicates that the short-wavelength band arises from singlet-state fluorescence, whereas the long-lived band is assigned to triplet-state phosphorescence populated through ISC [24]. Notably, compared to the pure APCl spectrum, the 500 nm phosphorescence signal in (AP)_2_ZnCl_4_ splits to reveal a distinct shoulder at 520 nm. This spectral evolution may be attributed to the rigidified hybrid lattice, which effectively restricts the motion of organic molecules and suppresses structural relaxation and inhomogeneous broadening. As a result, the vibronic features associated with triplet-state emission become more distinguishable, leading to the emergence of fine spectral structures in the phosphorescence band. Further evidence for the modified excited-state landscape is provided by the PLE spectra. When monitored at 500 nm, (AP)_2_ZnCl_4_ exhibits a dominant excitation peak at 360 nm (Figure 3b, gray curves), whereas APCl shows its strongest excitation around 430 nm (Figure 3a). This difference arises from their distinct hydrogen-bonding environments. In APCl, the protonated amino and carbonyl groups preferentially form intramolecular hydrogen bonds, which stabilize a more conjugated electronic structure and lower the excitation energy. By contrast, in (AP)_2_ZnCl_4_ the AP^+^ cations are immobilized through extensive intermolecular hydrogen bonding with the inorganic framework and lattice water molecules (Figure 1a,b), giving rise to a different electronic environment. This interpretation is further supported by the UV–vis absorption spectra in Figure 3a,b (dotted curves). APCl exhibits an absorption band at 355 nm, assigned to the *π* → π* transition of the benzenoid form, together with a red-shifted band at approximately 430 nm associated with the more conjugated quinonoid species generated through intramolecular proton transfer. In contrast, the hybrid crystal is dominated by the 355 nm absorption, while the 430 nm band is markedly suppressed, indicating that the hybrid lattice inhibits the formation of the quinonoid electronic state by replacing intramolecular hydrogen bonding with intermolecular interactions. Consequently, the excitation energy is shifted to higher energy in (AP)_2_ZnCl_4_. Benefiting from the rigid hybrid lattice and the enhanced SOC associated with the coordinated chloride environment, the photoluminescence quantum yield (PLQY) increases dramatically from 2% for APCl to 40% for (AP)_2_ZnCl_4_, reflecting suppressed non-radiative relaxation together with more efficient ISC [24].

### 3.3. Sb-Related Localized Emission and AP^+^-to-Sb Energy Transfer

Upon Sb introduction, a new broad emission band centered at approximately 620 nm emerges with a Stokes shift of about 260 nm (Figure 3c). The broad spectral profile and large Stokes shift are consistent with a localized/self-trapped excitonic assignment. The stereochemically active 5s^2^ lone pair of Sb^3+^ can favor local distortion and enhanced electron–phonon coupling [16,17,18,19,20,21,22,23], providing a plausible basis for Sb-related localization. Notably, the delayed PL peak positions of (AP)_2_ZnCl_4_:5%Sb are nearly identical to those of pristine (AP)_2_ZnCl_4_ (Figure 3f). Corresponding delayed-emission contour and composition-dependent spectral data are provided in Figures S3 and S4. These observations indicate that the native AP^+^-derived phosphorescence color remains essentially unchanged while the added Sb-related channel primarily modifies the prompt-emission response.

**Figure 4:**
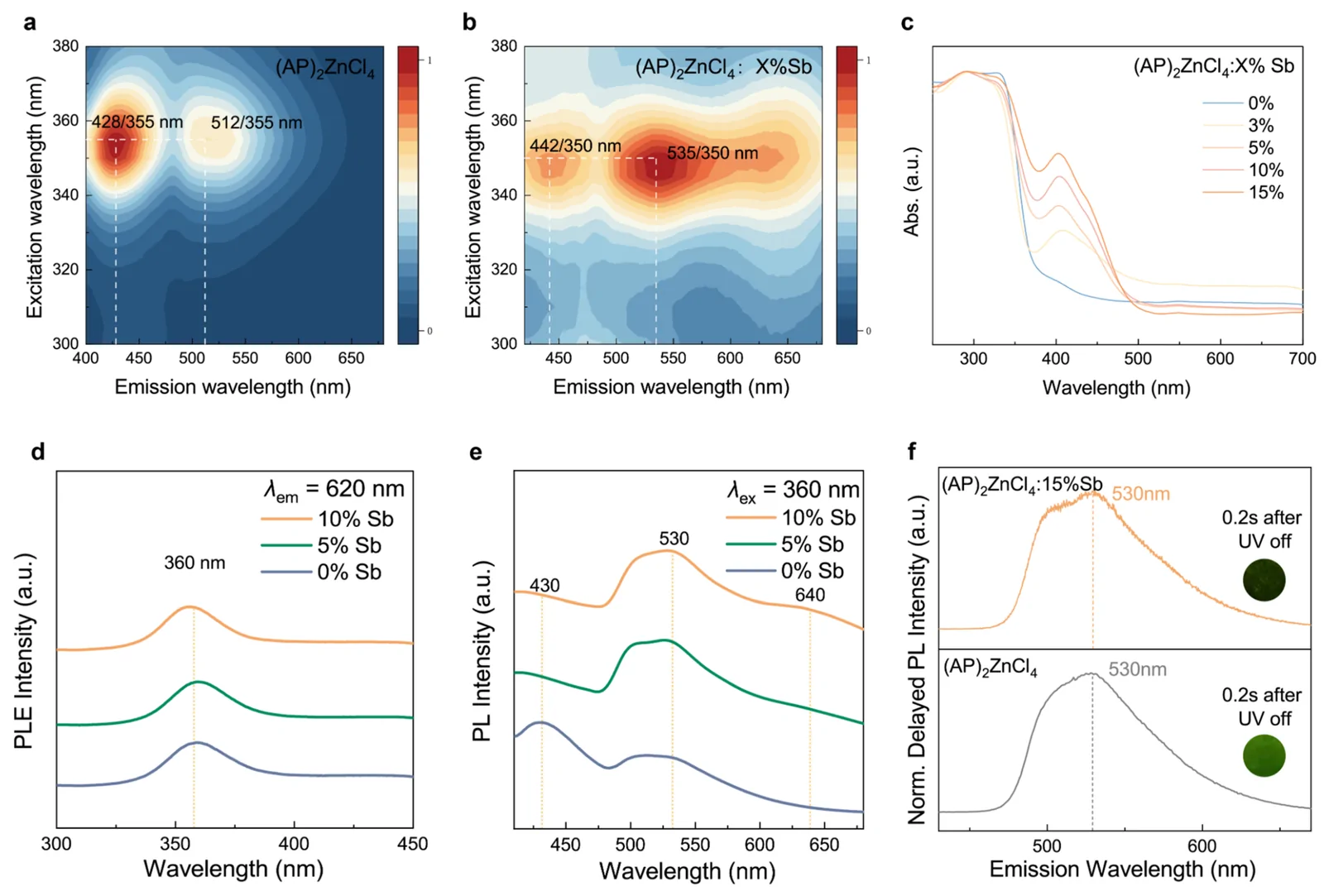
Excitation-dependent optical properties of pristine and nominally Sb-modified (AP)_2_ZnCl_4_. (**a**,**b**) Excitation–emission intensity maps of pristine (AP)_2_ZnCl_4_ and the Sb-modified sample, respectively. Each matrix point corresponds to an experimentally acquired measurement point; no smoothing, resampling, interpolation to additional data points, or merging of neighboring measurements was applied, and the apparent continuity arises from contour rendering. (**c**) Ultraviolet–visible absorption spectra of (AP)_2_ZnCl_4_:*x*%Sb (*x* = 0, 3, 5, 10, and 15). (**d**) PLE spectra monitored at 620 nm and (**e**) prompt PL spectra recorded under 360 nm excitation for *x* = 0, 5, and 10. (**f**) Normalized delayed PL spectra and corresponding afterglow photographs of pristine (AP)_2_ZnCl_4_ and (AP)_2_ZnCl_4_:15%Sb, recorded 0.2 s after removal of the ultraviolet excitation.

To further elucidate the emission mechanism, excitation-dependent 3D prompt PL spectroscopy was performed. For pristine (AP)_2_ZnCl_4_, the emission-peak positions and overall excitation-dependent spectral profiles remain essentially unchanged (Figure 4a; Figure S5), supporting their origin from the intrinsic excited-state relaxation of AP^+^ cations. In contrast, the spectra of the nominally Sb-modified sample retain the characteristic organic emission while exhibiting an additional broad band centered near 620 nm (Figure S6), consistent with an Sb-related localized/self-trapped emissive state.

The UV–vis diffuse-reflectance spectra reveal an Sb-related absorption feature near 430 nm after Sb introduction (Figure 4c), assigned to the spin-allowed ^1^S_0_ → ^3^P_1_ transition of Sb^3+^ [16,17,18,19,20,21,22,23]. Its intensity increases with nominal Sb content. By contrast, the 620 nm excitation response is dominated by the host-related excitation region near 360 nm, and direct excitation of (AP)_2_ZnCl_4_:5%Sb at 435 nm produces no distinct 620 nm band above the background (Figure S7). Thus, direct excitation of the resolved Sb-related transition is unlikely to be the dominant source of the 620 nm emission under 360 nm excitation. The emission spectrum of pristine (AP)_2_ZnCl_4_ overlaps the Sb-related absorption region, providing an energetic basis for sensitization [28,29]. Consistently, increasing nominal Sb content suppresses the AP^+^-centered 430–435 nm fluorescence while enhancing the broad 620 nm emission (Figure 4e; Figure S8). Together with the donor-lifetime shortening discussed below, these reciprocal spectral changes support AP^+^-to-Sb energy transfer. The corresponding composition-dependent delayed-emission profiles are compared in Figure S9.

Finally, to evaluate whether the 620 nm band could instead arise from trap-mediated or nonlinear free-carrier recombination, its excitation-power dependence was analyzed using the following power-law relationships:

(1) $$I=aP^{k}$$

(2) logI=loga+klogP

where *I* is the integrated long-wavelength emission intensity, *P* is the excitation power, *a* is a proportionality constant, and *k* is the fitted exponent. The double-logarithmic fit yields *k* = 0.96 with *R*^2^ = 0.997, and no evident saturation is observed at higher excitation powers over the measured range (Figure S10). This approximately first-order, one-photon response disfavors a trap-filling-dominated mechanism and a strongly nonlinear free-carrier recombination process as the dominant origin within the measured range, although minor trapping or charge-transfer contributions cannot be completely excluded. Together with the direct-excitation control, concentration dependence, and time-resolved data, these results support an Sb-related localized/self-trapped excitonic emission as the predominant assignment for the 620 nm band. More broadly, spatial and energetic distributions of trap states can strongly influence carrier behavior in metal-halide perovskites, underscoring the importance of distinguishing trap-mediated relaxation from intrinsic localized-emission pathways [30].

### 3.4. Excited-State Dynamics and Photophysical Mechanism

**Figure 5:**
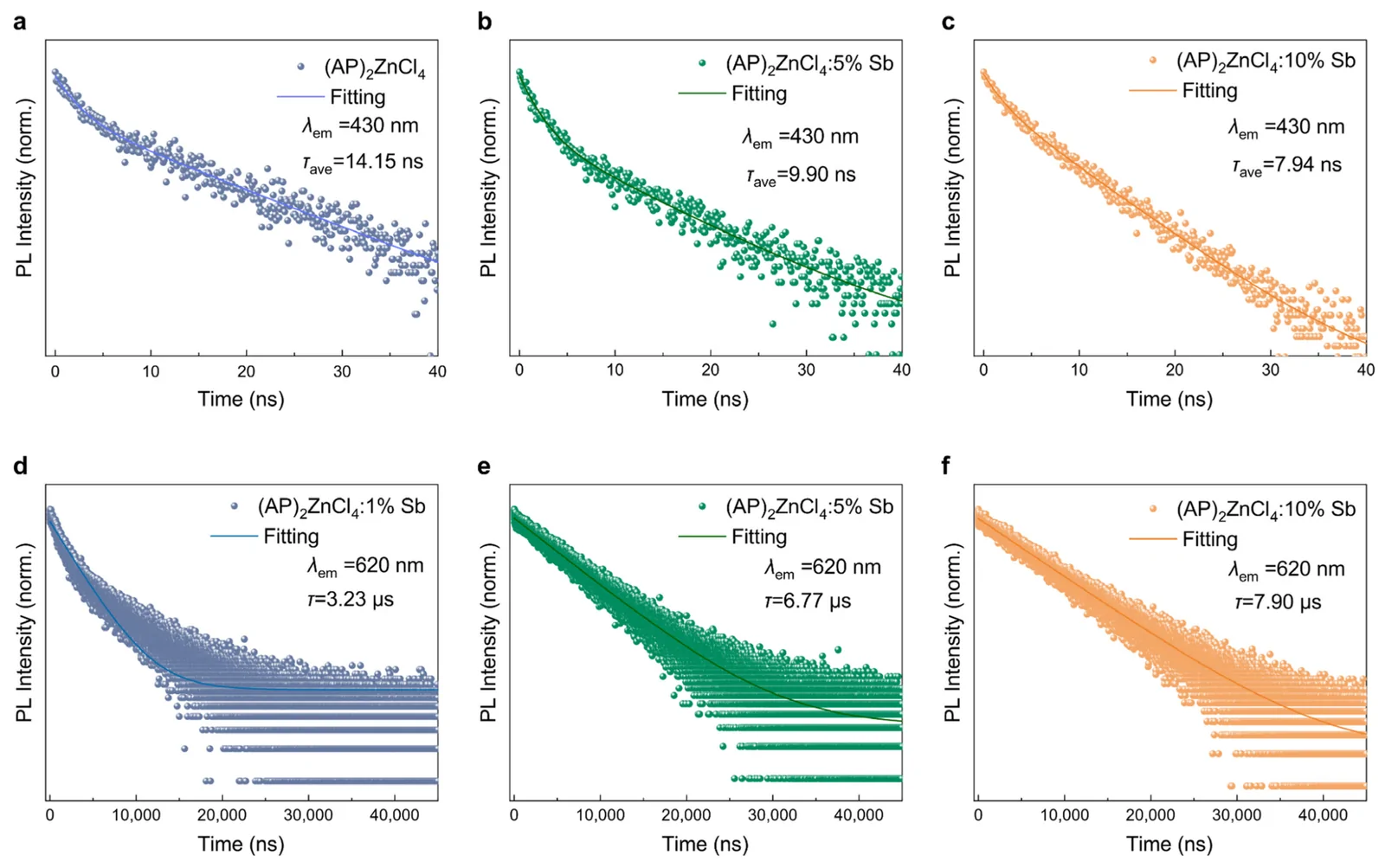
Photophysical dynamics of (AP)_2_ZnCl_4_:*x*%Sb under 360 nm excitation. (**a**–**c**) TRPL decay curves monitored at 430 nm for pristine (AP)_2_ZnCl_4_, (AP)_2_ZnCl_4_:5%Sb, and (AP)_2_ZnCl_4_:10%Sb, respectively, fitted using a biexponential decay model, with intensity-weighted average lifetimes of 14.15, 9.90, and 7.94 ns. (**d**–**f**) TRPL decay curves monitored at 620 nm for (AP)_2_ZnCl_4_:1%Sb, (AP)_2_ZnCl_4_:5%Sb, and (AP)_2_ZnCl_4_:10%Sb, respectively, fitted using a biexponential decay model, with intensity-weighted average lifetimes of 3.23, 6.77, and 7.90 μs. Symbols represent the experimental data and solid lines represent the fitted curves.

Time-resolved photoluminescence under 360 nm excitation provides kinetic support for AP^+^-to-Sb energy transfer. The average 430 nm lifetime decreases systematically from 14.15 ns in pristine (AP)_2_ZnCl_4_ to 9.90, 7.94, and 7.85 ns at 5%, 10%, and 15% Sb, respectively (Figure 5a–c; Table S5), showing that Sb introduction opens an additional decay channel for the AP^+^ S_1_ state. Meanwhile, the Sb-related 620 nm emission displays microsecond decay, with average lifetimes of 3.23, 6.77, 7.90, and 6.95 μs at 1%, 5%, 10%, and 15% Sb, respectively (Figure 5d–f; Table S3). Together with the excitation-dependent spectra and reciprocal composition-dependent emission changes, the donor-lifetime shortening is consistent with transfer from the organic S_1_ state to Sb-related localized emissive states. A lifetime-derived apparent transfer efficiency was estimated using Equation (3) [28], where *τ_ave_* and *τ* are the average 430 nm fluorescence lifetimes of the doped and undoped samples, respectively. The resulting apparent efficiencies are 30.0%, 43.9%, and 44.5% for 5%, 10%, and 15% Sb (Table S5). The increasing apparent transfer fraction with Sb^3+^ content is consistent with increasingly efficient depopulation of the organic singlet channel in the presence of Sb-related acceptor states.

(3) $$\Phi =1-\frac{\tau_{ave}}{\tau }$$

Notably, as the nominal Sb content increases from 5% to 10%, the average lifetime of the 620 nm emission increases from 6.77 to 7.90 μs, accompanied by an increase in PLQY from 62.4% to 75.6%. At 15% Sb, however, the lifetime and PLQY decrease to 6.95 μs and 61.5%, respectively (Table S6). The 10% sample therefore exhibits the most favorable balance between population of the Sb-related emissive state and suppression of nonradiative relaxation. The simultaneous decrease in lifetime and PLQY at 15% Sb is consistent with the onset of concentration-dependent quenching, potentially arising from increased dopant–dopant interactions and additional nonradiative relaxation pathways at higher loading. Related work on tin-lead perovskite nanocrystals has shown that energetic disorder and carrier–phonon interactions can jointly modulate optical properties and recombination dynamics, highlighting the sensitivity of excited-state relaxation to the local structural environment [31].

The lifetime of the delayed 525 nm emission varies nonmonotonically, increasing from 71.0 ms in the 1% Sb sample to 88.7 and 95.2 ms in the 5% and 10% Sb samples, respectively, before decreasing to 85.3 ms at 15% Sb (Figure S9 and Table S4). The delayed spectral position remains essentially unchanged across the series, indicating that the AP^+^-derived RTP pathway remains operative after Sb introduction. Importantly, the 525 nm decay lifetime reflects the triplet-state decay rate rather than its absolute population; therefore, the longer lifetime at moderate Sb content is not interpreted as evidence for a larger triplet population. Instead, the nonmonotonic trend is consistent with moderate Sb introduction modifying the local relaxation environment while preserving long-lived RTP, whereas higher loading introduces additional nonradiative loss. Overall, the concentration-dependent data support redistribution among the AP^+^ singlet, Sb-related localized, and AP^+^ triplet channels rather than being simply quenched across all emissive pathways.

**Figure 6:**
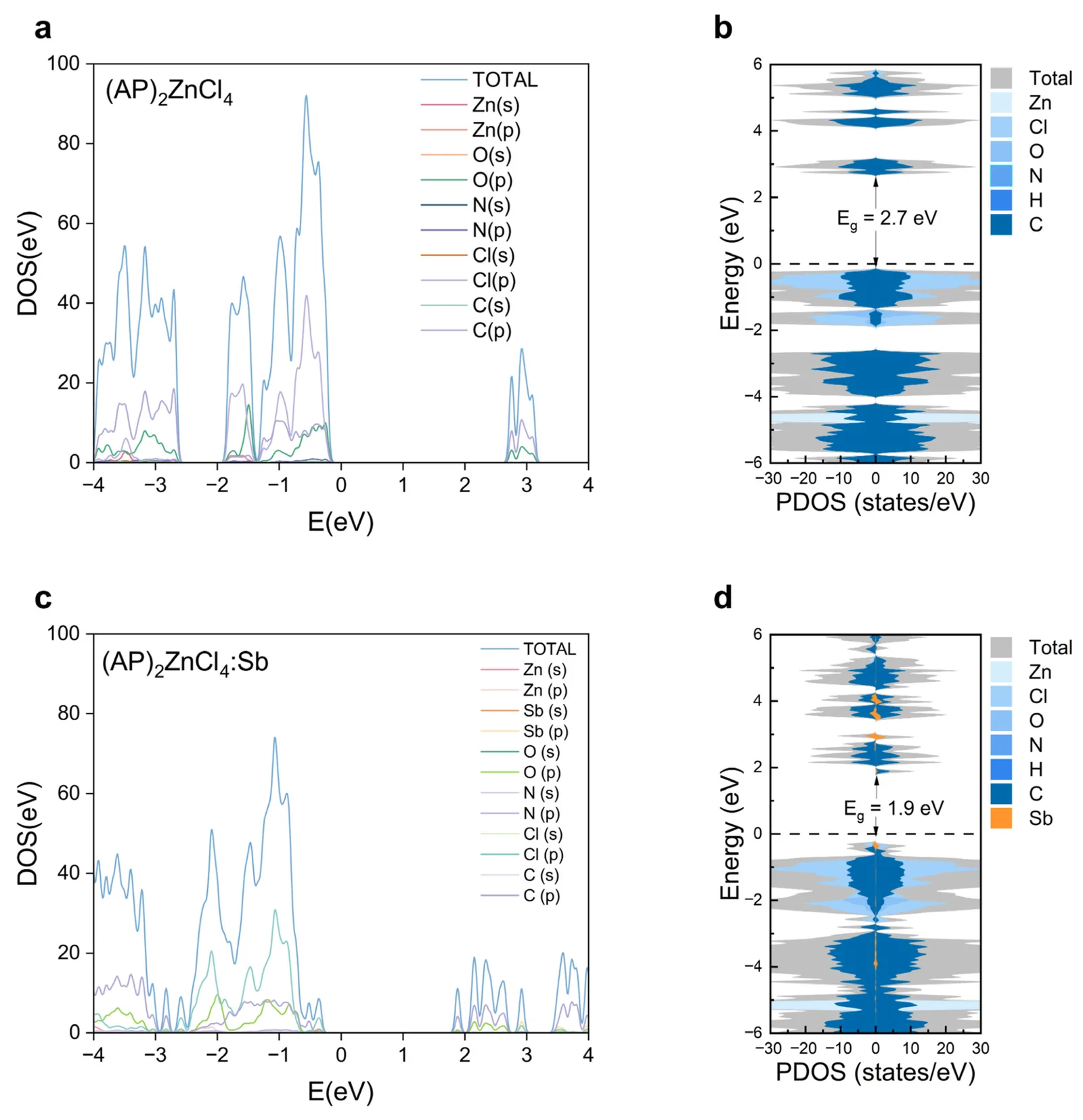
DFT-derived ground-state electronic structures of pristine and Sb-containing (AP)_2_ZnCl_4_. (**a**,**b**) Orbital-resolved DOS and element-resolved PDOS of pristine (AP)_2_ZnCl_4_, respectively; the calculated bandgap in (**b**) is approximately 2.7 eV. (**c**,**d**) Corresponding DOS and PDOS of the Sb-containing model; the calculated bandgap in (**d**) is approximately 1.9 eV.

To provide ground-state electronic-structure context for the composition-dependent photophysics, DFT calculations were used to examine the band structures and projected density of states (PDOS) of pristine and Sb-containing (AP)_2_ZnCl_4_. In pristine (AP)_2_ZnCl_4_, the valence-band maximum (VBM) and conduction-band minimum (CBM) are dominated mainly by O-p and N-p orbitals of the AP^+^ cations, with additional Cl-3p contributions, whereas Zn contributes negligibly near the band edges because of its closed-shell d^10^ configuration. The participation of Cl-3p states is consistent with an inorganic chloride environment capable of enhancing spin–orbit coupling. The calculated bandgap of the pristine model is approximately 2.7 eV (Figure 6a,b). These calculations are used to describe the ground-state electronic structure rather than to establish an excited-state transfer or self-trapping pathway.

For the charge-compensated Sb-containing model, the PDOS shows Sb-induced states near the band edges, with Sb-s contribution near the VBM and Sb-p/Cl-p hybridized character near the CBM (Figure 6c,d), reducing the calculated bandgap from approximately 2.7 to 1.9 eV. The CBM is spatially localized around the disphenoidal [SbCl_4_]^−^ unit, indicating that Sb incorporation creates a localized electronic environment capable of hosting an Sb-related excitonic state. These Sb-related states provide new excited-state relaxation pathways, which agrees well with the experimentally observed emergence of broad emission from STEs centered at 620 nm. Furthermore, compared with the pristine system, the flatter dispersion of the VBM and CBM in Sb^3+^-doped (AP)_2_ZnCl_4_ indicates reduced electronic bandwidth and enhanced carrier localization. The localized nature of these Sb-derived states facilitates electron–phonon coupling and lattice distortion, which are essential characteristics for the formation of STEs. Therefore, the DFT calculations reveal that Sb^3+^ incorporation not only modifies the electronic band structure but also creates localized excited-state environments, providing a theoretical basis for the Sb-induced emission from STEs.

**Figure 7:**
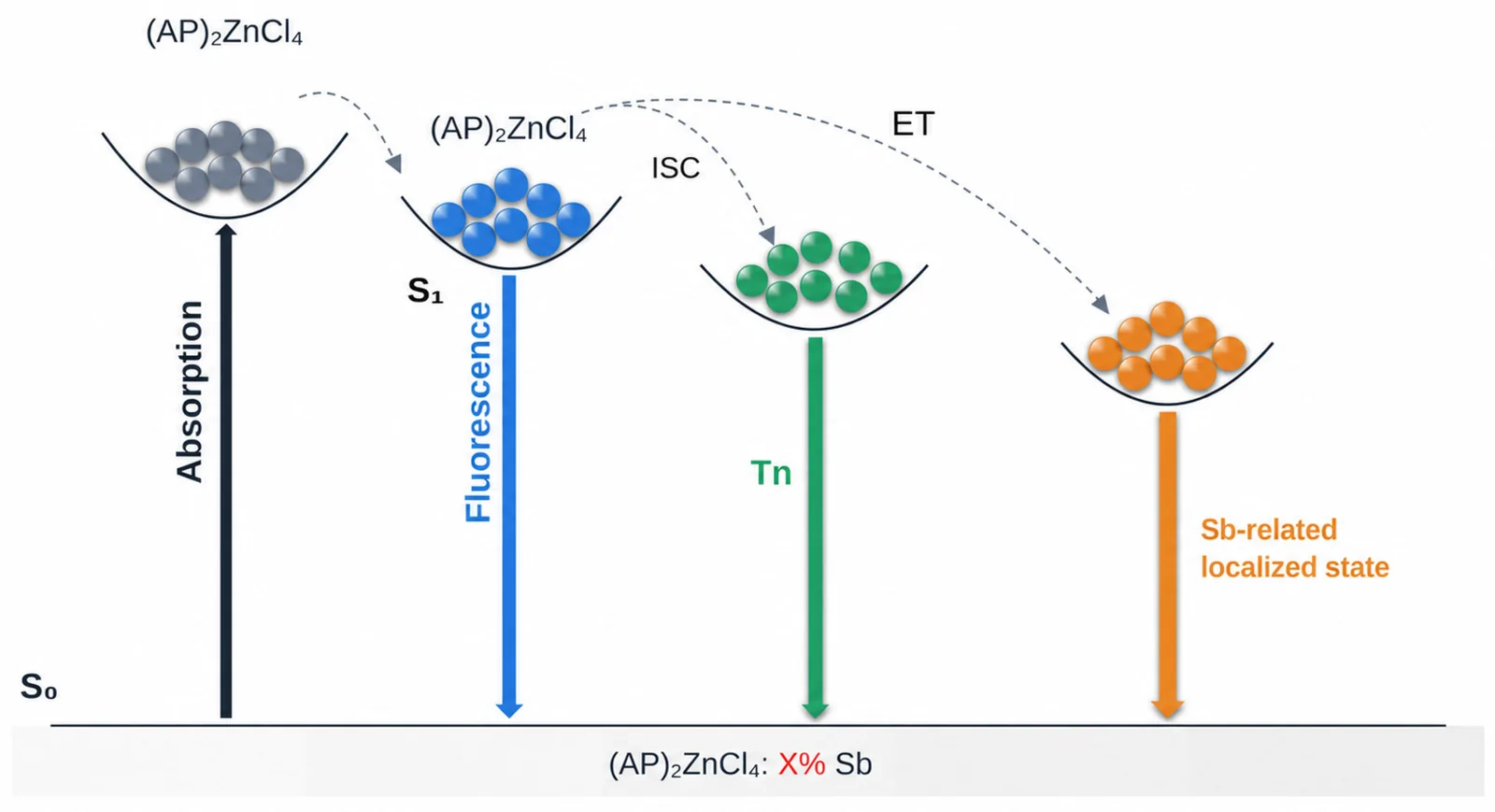
Proposed photophysical mechanism of pristine and nominally Sb-modified (AP)_2_ZnCl_4_. The schematic illustrates photoexcitation and prompt fluorescence from the AP^+^ singlet manifold, ISC followed by AP^+^-derived phosphorescence, and an additional AP^+^-to-Sb energy-transfer pathway that populates Sb-related localized/self-trapped excitonic states after Sb introduction. The diagram represents a working mechanism supported by the combined spectroscopic and kinetic evidence, rather than proof of a unique Förster process or a directly observed excited-state structure. Black, blue, green, and orange denote absorption, fluorescence, triplet/RTP relaxation, and Sb-related emission, respectively.

Based on the above experimental results, with DFT used as supportive ground-state context, the proposed photophysical mechanism of (AP)_2_ZnCl_4_:*x*%Sb is summarized in Figure 7. Upon photoexcitation, AP^+^ chromophores are promoted from S_0_ to higher singlet states and relax to S_1_ (Path 1). In the pristine crystal, S_1_ excitons either return to the ground state through prompt fluorescence or undergo intersystem crossing to populate triplet states that give rise to long-lived AP^+^-derived RTP (Path 2). After Sb introduction, the combined spectral and lifetime evidence supports an additional AP^+^-to-Sb transfer channel from the organic S_1_ manifold to Sb-related localized/self-trapped emissive states [28,29], which subsequently produce the broad orange-red emission (Path 3). The scheme therefore represents an evidence-supported working model for the coexistence of prompt fluorescence, Sb-related localized emission, and persistent RTP.

### 3.5. Time-Gated Optical Information Encoding and Decoding

**Figure 8:**
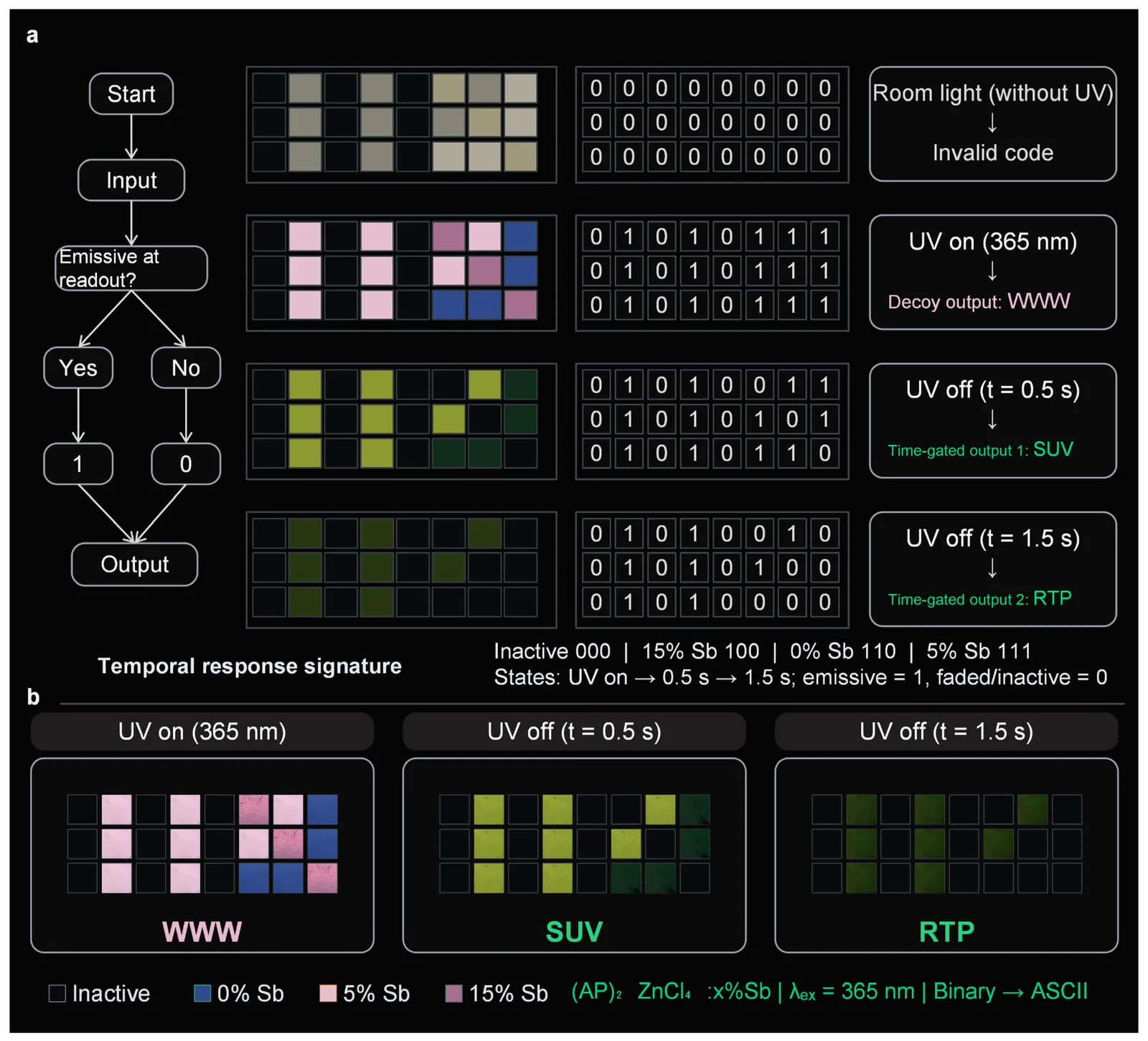
Proof-of-concept time-gated optical encoding and sequential decoding using (AP)_2_ZnCl_4_:*x*%Sb luminescent arrays. (**a**) Schematic illustration of the binary encoding and temporal decoding procedure. Three selected readout states were defined as UV on, 0.5 s after excitation removal, and 1.5 s after excitation removal. Emissive pixels were assigned as 1, whereas sufficiently faded or inactive pixels were assigned as 0. Accordingly, inactive, 15% Sb, 0% Sb, and 5% Sb pixels exhibit temporal response signatures of 000, 100, 110, and 111, respectively. (**b**) Corresponding photographs of the 3 × 8 array at the three selected readout states, yielding sequential outputs of “WWW”, “SUV”, and “RTP”. The corresponding semi-quantitative ROI analysis is provided in Figure S11. *λ*_ex_ = 365 nm. In the composition legend, blue, light-pink, purple, and outlined dark symbols denote 0% Sb, 5% Sb, 15% Sb, and inactive sites, respectively.

Taking advantage of the composition-dependent afterglow persistence of (AP)_2_ZnCl_4_:*x*%Sb, a proof-of-concept time-gated optical encoding array was constructed using inactive sites and crystals with nominal *x* = 0, 5, and 15. Three selected readout states were defined: UV on, 0.5 s after removal of the excitation source, and 1.5 s after removal. At each selected state, an emissive pixel was assigned a binary value of 1, whereas a sufficiently faded or inactive pixel was assigned 0. Under this rule, the inactive, 15% Sb, 0% Sb, and 5% Sb pixels exhibit temporal response signatures of 000, 100, 110, and 111, respectively (Figure 8a). These pixel types were spatially arranged into a 3 × 8 array containing 24 binary pixels. Under continuous 365 nm irradiation, all three rows produce 01010111, corresponding to the ASCII character “W” and yielding the initial output “WWW”.

After the UV source is removed, the composition-dependent persistence of the encoded pixels produces sequential readout. At 0.5 s, the three rows become 01010011, 01010101, and 01010110, corresponding to “SUV”; at 1.5 s, the remaining emissive pixels generate 01010010, 01010100, and 01010000, corresponding to “RTP” (Figure 8b). A semi-quantitative ROI analysis of photographs acquired under identical imaging conditions further supports the distinct temporal persistence of the 0%, 5%, and 15% Sb pixel types (Figure S11). These data are used to support the contrast at the selected readout states rather than to define a universal binary threshold or temporal tolerance window. The demonstration therefore establishes a proof-of-concept sequential time-gated optical encoding/decoding process, rather than a fully validated multidimensional encryption platform.

## 4. Conclusions

In summary, Sb introduction effectively regulates the excited-state dynamics of the 0D hybrid metal halide (AP)_2_ZnCl_4_, introducing a broad Sb-related localized/self-trapped excitonic emission while preserving the intrinsic AP^+^-derived RTP. Spectroscopic and time-resolved measurements indicate an additional singlet-state relaxation pathway involving Sb-related localized states, enabling the coexistence of tunable prompt emission and persistent phosphorescence. Ground-state DFT supports Sb-induced band-edge modification and localization but is not treated as proof of excited-state transfer or self-trapping. By exploiting these composition- and lifetime-dependent optical responses, a proof-of-concept time-gated optical encoding/decoding scheme with sequential “WWW”, “SUV”, and “RTP” outputs was demonstrated. These results highlight the potential of dopant-mediated excited-state regulation in 0D hybrid metal halides for dynamic optical information encoding. Nevertheless, the microscopic excited-state structure and self-trapping process of the Sb-related emissive state remain unresolved. Further excited-state and local-structure studies are needed to clarify the mechanism and advance dynamic optical information encoding.

## Data Availability

The original contributions presented in this study are included in the article/Supplementary Material. Further inquiries can be directed to the corresponding authors.

## Supplementary Materials

The following supporting information can be downloaded at: https://www.mdpi.com/article/10.3390/nano16171121/s1; Figure S1: SEM image and EDS elemental maps of the nominal 5% Sb sample; Figure S2: Phosphorescence excitation–emission contour map of the undoped (AP)_2_ZnCl_4_ host; Figure S3: Phosphorescence excitation–emission contour map of (AP)_2_ZnCl_4_:15%Sb; Figure S4: Delayed photoluminescence spectra of (AP)_2_ZnCl_4_:*x*%Sb; Figure S5: Normalized excitation-dependent photoluminescence spectra of undoped (AP)_2_ZnCl_4_; Figure S6: Normalized excitation-dependent photoluminescence spectra of the nominally Sb-modified sample; Figure S7: Prompt PL spectrum of (AP)_2_ZnCl_4_:5%Sb under 435 nm excitation; Figure S8: Normalized photoluminescence spectra of (AP)_2_ZnCl_4_:*x*%Sb; Figure S9: Time-resolved phosphorescence decay profiles monitored at 525 nm; Figure S10: Power-dependent prompt PL spectra and double-logarithmic analysis of the Sb-related long-wavelength emission; Figure S11: Semi-quantitative ROI analysis of composition-dependent afterglow persistence; Figure S12: Experimental repeatability of the delayed photoluminescence spectra of pristine (AP)_2_ZnCl_4_; Table S1: Single-crystal X-ray diffraction data of APCl; Table S2: Single-crystal X-ray diffraction data of (AP)_2_ZnCl_4_·H_2_O; Table S3: Photoluminescence lifetime of (AP)_2_ZnCl_4_:*x*%Sb monitored at 620 nm; Table S4: Delayed photoluminescence lifetime parameters monitored at 525 nm; Table S5: Calculated apparent energy-transfer efficiency monitored at 430 nm; Table S6: PLQY of (AP)_2_ZnCl_4_:*x*%Sb.
