# Supplementary material for: Integrating Fluorescence from Self-Trapped Excitons and Phosphorescence in Zero-Dimensional Metal Halides for Time-Resolved Dynamic Information Encryption
Source: Nanomaterials (Basel). 2026 Sep 7;16(17):1121. doi: 10.3390/nano16171121 (PMC13567518; doi:10.3390/nano16171121)
Supplement: Supplementary file 1 [file nanomaterials-16-01121-s001.zip › nanomaterials-4532374-supplementary.pdf]

Supporting Information

Integrating Fluorescence from Self-Trapped Excitons and Phosphorescence in Zero-Dimensional Metal Halides for Time-Resolved Dynamic Information Encryption

Xiang Zhu[1]†, Lei Li[1]†, Fei Wen[1], Yu Wang[1], Yangbin Xu[1], Zhixuan Wang[1], Cuixia You[1], Qingchun Chen[1], Lingling Xu[1], Jiansong Ye[1], Jiaxing Song[1], Nengchao Qiu[1], Yanxing Feng[4], Tingwei He[2]*, Hai Jia[3]*, and Quanlin Chen[1]*

*† These authors contributed equally to this work. * Corresponding authors.*

Supplementary Tables

Table S1. Single-crystal X-ray diffraction data of APCl.

**Empirical formula**	**$C_8H_{10}ClNO$**
Formula weight	171.62
Temperature / K	193
Crystal system	monoclinic
Space group	$P2_1/c$
a / Å	4.8984
b / Å	15.8013
c / Å	10.8025
α / °	90
β / °	102.614
γ / °	90
Volume / Å^3	815.94
Z	4
ρ_{calc} / g·cm^{-3}	1.397
F(000)	360
wR_2 (reflections)	0.1503

Table S2. Single-crystal X-ray diffraction data of $(AP)_2ZnCl_4\cdot H_2O$.

**Empirical formula**	**$C_{16}H_{22}Cl_4N_2O_3Zn$**
Formula weight	497.52
Temperature / K	296.15
Crystal system	monoclinic
Space group	$P2_1/n$
a / Å	7.308
b / Å	16.775
c / Å	17.665

Empirical formula	$C_{16}H_{22}Cl_4N_2O_3Zn$
α / °	90
β / °	101.133
γ / °	90
Volume / Å^3	2124.7
Z	4
ρ_{calc} / g·cm^{-3}	1.555
F(000)	1016
wR_2 (reflections)	0.1465

Table S3. PL lifetime of $(AP)_2ZnCl_4$:x%Sb monitored at 620 nm.

	$(AP)_2ZnCl_4$:1%Sb	$(AP)_2ZnCl_4$:5%Sb	$(AP)_2ZnCl_4$:10%Sb	$(AP)_2ZnCl_4$:15%Sb
τ_{ave} (μs)	3.23	6.77	7.90	6.95

Table S4. Delayed PL lifetime parameters of $(AP)_2ZnCl_4$:x%Sb monitored at 525 nm.

	$(AP)_2ZnCl_4$:1%Sb	$(AP)_2ZnCl_4$:5%Sb	$(AP)_2ZnCl_4$:10%Sb	$(AP)_2ZnCl_4$:15%Sb
τ_1 (ms)	21.09	17.20	17.54	17.92
τ_2 (ms)	86.10	107.17	109.33	98.56
τ_{ave} (ms)	71.00	88.7	95.2	85.3

Table S5. Calculated apparent energy-transfer efficiency for $(AP)_2ZnCl_4$:x%Sb monitored at 430 nm.

	$(AP)_2ZnCl_4$	$(AP)_2ZnCl_4$:5%Sb	$(AP)_2ZnCl_4$:10%Sb	$(AP)_2ZnCl_4$:15%Sb
τ_1 (ns)	2.21	1.94	1.81	1.8
τ_2 (ns)	15.67	11.89	8.62	8.53
τ_{ave} (ns)	14.15	9.90	7.94	7.85
Φ	/	30.0%	43.9%	44.5%

Table S6. PLQY for $(AP)_2ZnCl_4$:x%Sb.

	$(AP)_2ZnCl_4$	$(AP)_2ZnCl_4$:5%Sb	$(AP)_2ZnCl_4$:10%Sb	$(AP)_2ZnCl_4$:15%Sb
PLQY	40.0%	62.4%	75.6%	61.5%

Supplementary Figures

SEM–EDS characterization of the nominal 5% Sb sample

SEM–EDS elemental mapping confirms the presence of Sb across the analyzed region together with Zn and Cl, without obvious large-scale Sb-rich segregation. These data are used as compositional and micrometer-scale distribution evidence and do not establish a specific crystallographic Sb site or local coordination environment.

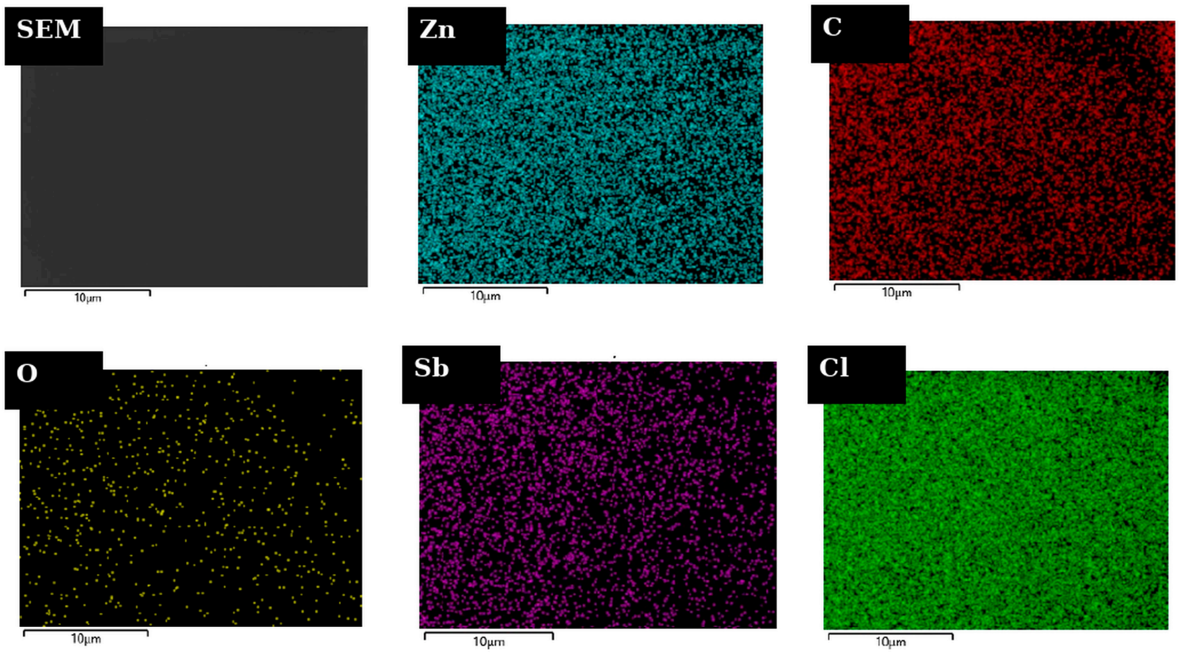

Figure S1. SEM image and corresponding EDS elemental maps of the nominal 5% Sb sample. Zn, C, O, Sb, and Cl maps were acquired from the same analyzed region (scale bar: 10 μm). The Sb signal is distributed across the mapped area without obvious large-scale Sb-rich segregation. The mapping is used to support the presence and micrometer-scale distribution of Sb rather than to assign a specific crystallographic site or local coordination environment.

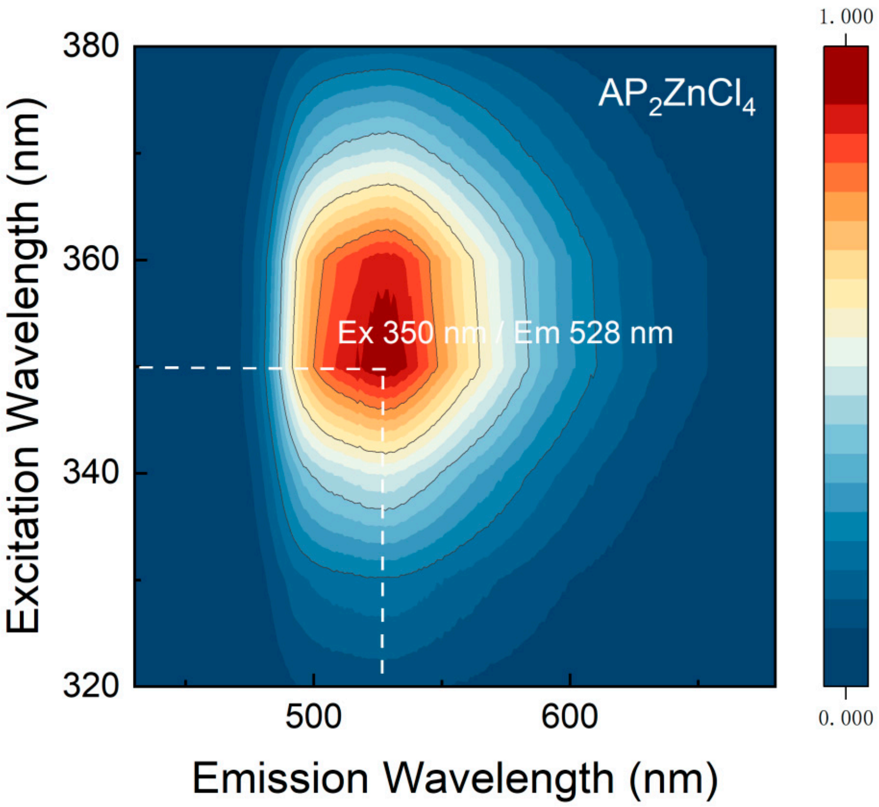

Figure S2. Phosphorescence excitation–emission contour map of the undoped $(AP)_2$ $ZnCl_4$ host. Relative emission intensity is plotted as a function of excitation and emission wavelength; the dashed guides mark the intensity maximum at $\lambda ex = 350$ nm and $\lambda em = 528$ nm.

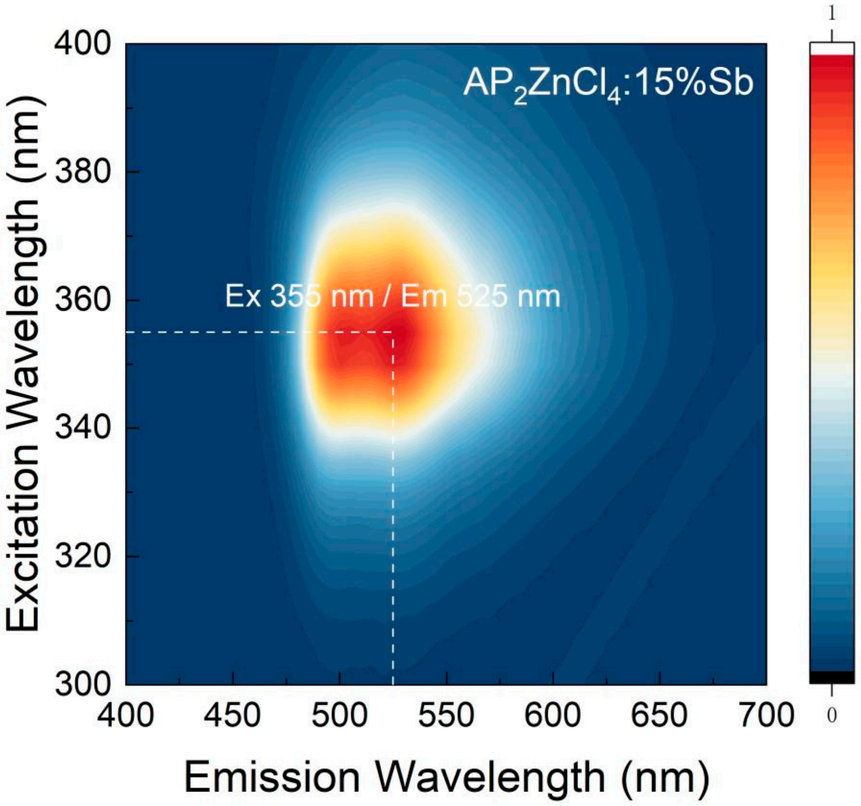

Figure S3. Phosphorescence excitation–emission contour map of the (AP)$_2$ZnCl$_4$:15%Sb sample. Relative emission intensity is plotted as a function of excitation and emission wavelength; the dashed guides identify the maximum at λex = 355 nm and λem = 525 nm.

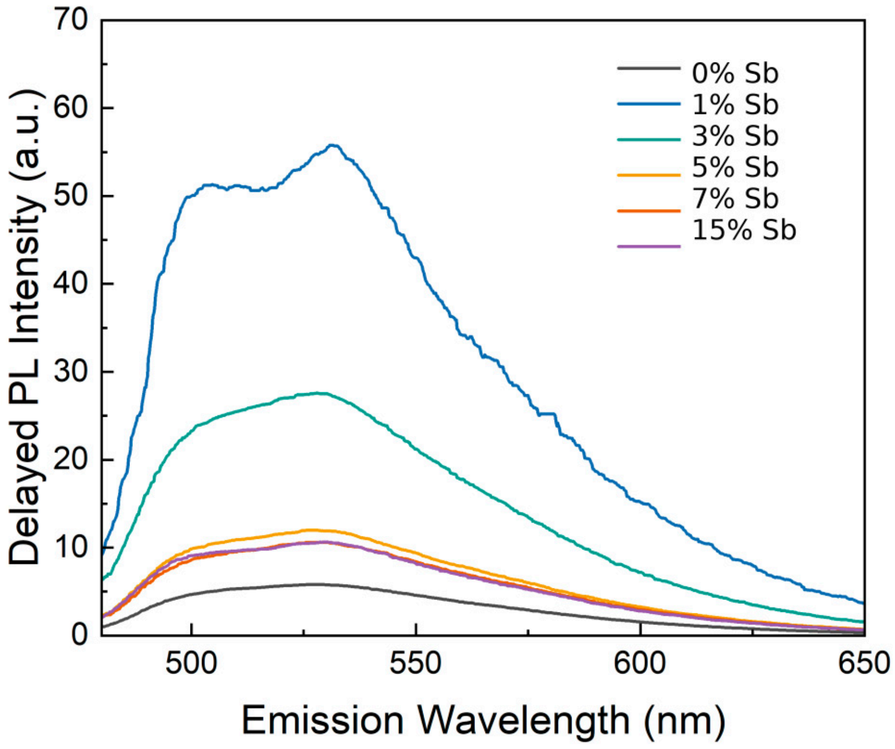

Figure S4. Delayed photoluminescence emission spectra of (AP)$_2$ZnCl$_4$:x%Sb (x = 0, 1, 3, 5, 7, and 15) recorded from 480 to 650 nm. Delayed-PL intensity is reported in arbitrary units.

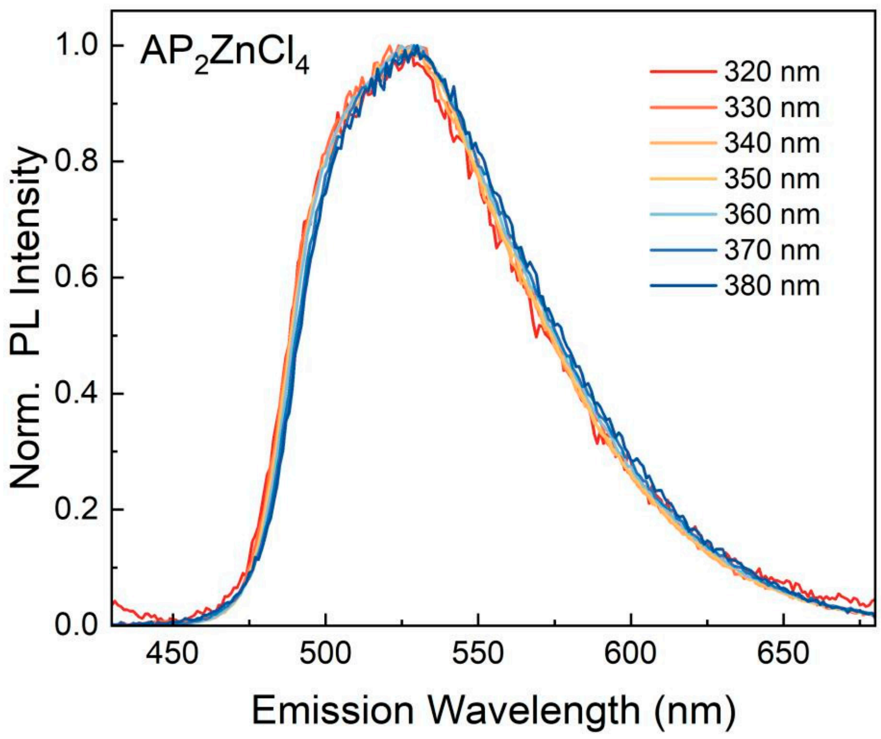

Figure S5. Normalized excitation-dependent photoluminescence spectra of undoped (AP)$_2$ $ZnCl_4$. Emission spectra recorded at excitation wavelengths from 320 to 380 nm are normalized to facilitate comparison of their spectral profiles.

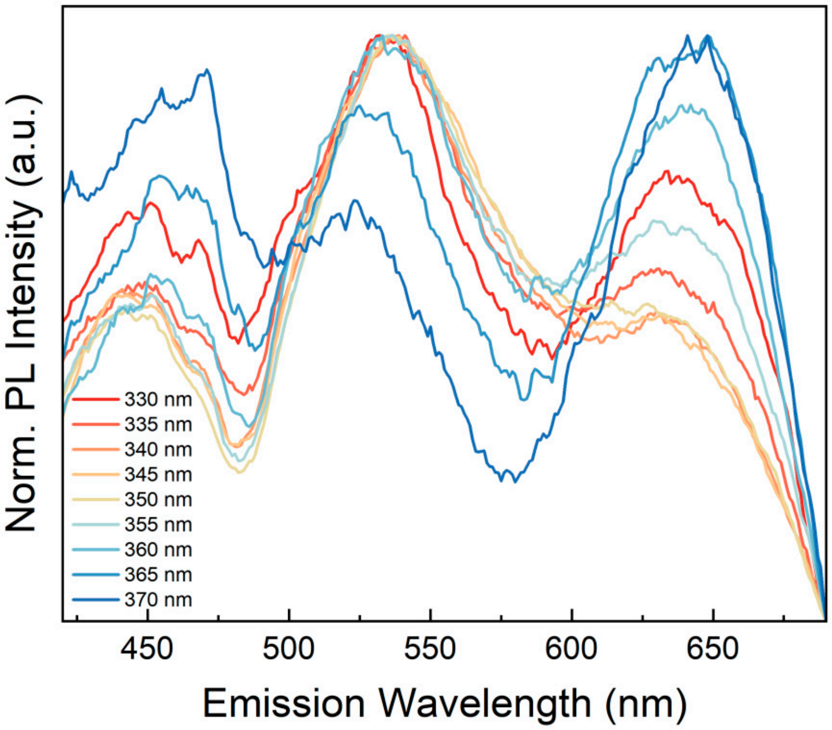

Figure S6. Normalized excitation-dependent photoluminescence spectra of the nominally Sb-modified (AP)$_2$ $ZnCl_4$ sample. Emission spectra recorded at excitation wavelengths from 330 to 370 nm are normalized to compare the excitation-dependent spectral response.

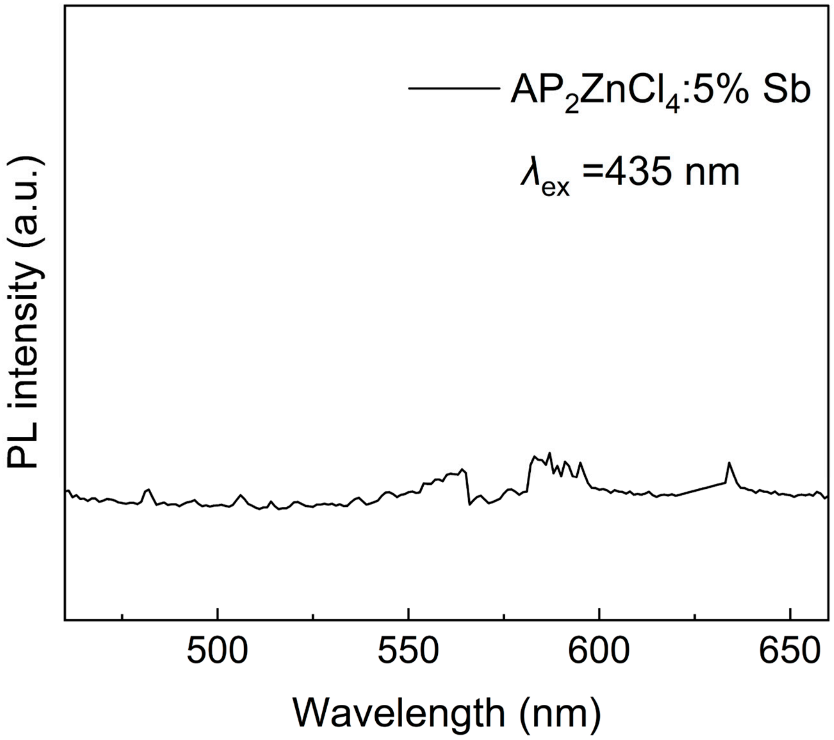

Figure S7. Prompt PL spectrum of $(AP)_2ZnCl_4$:5%Sb recorded under 435 nm excitation. No distinct 620 nm emission band is resolved above the background.

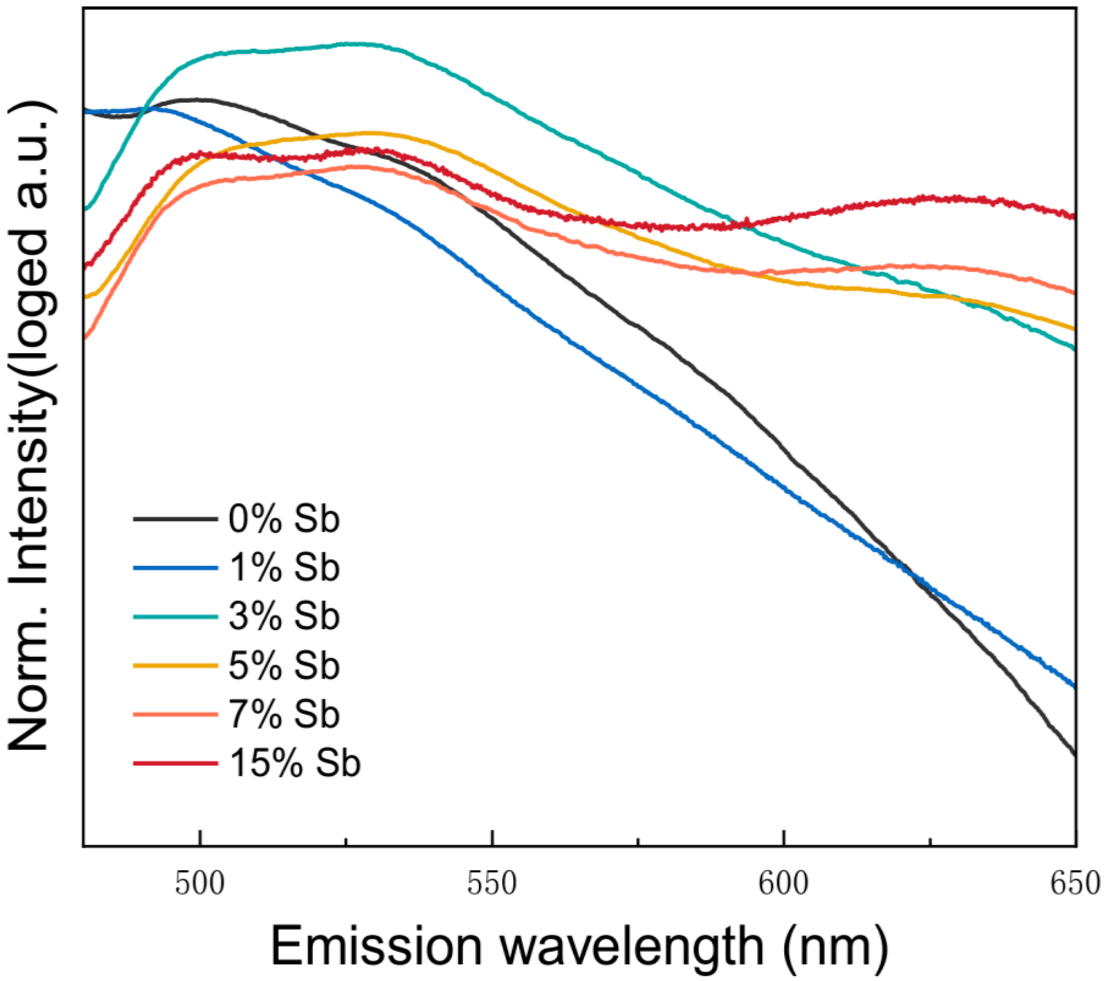

Figure S8. Normalized photoluminescence emission spectra of (AP)$_2$ZnCl$_4$:x%Sb (x = 0, 1, 3, 5, 7, and 15) recorded from 480 to 650 nm. The normalized intensities are displayed on a logarithmic scale to compare the spectral profiles across the Sb-composition series.

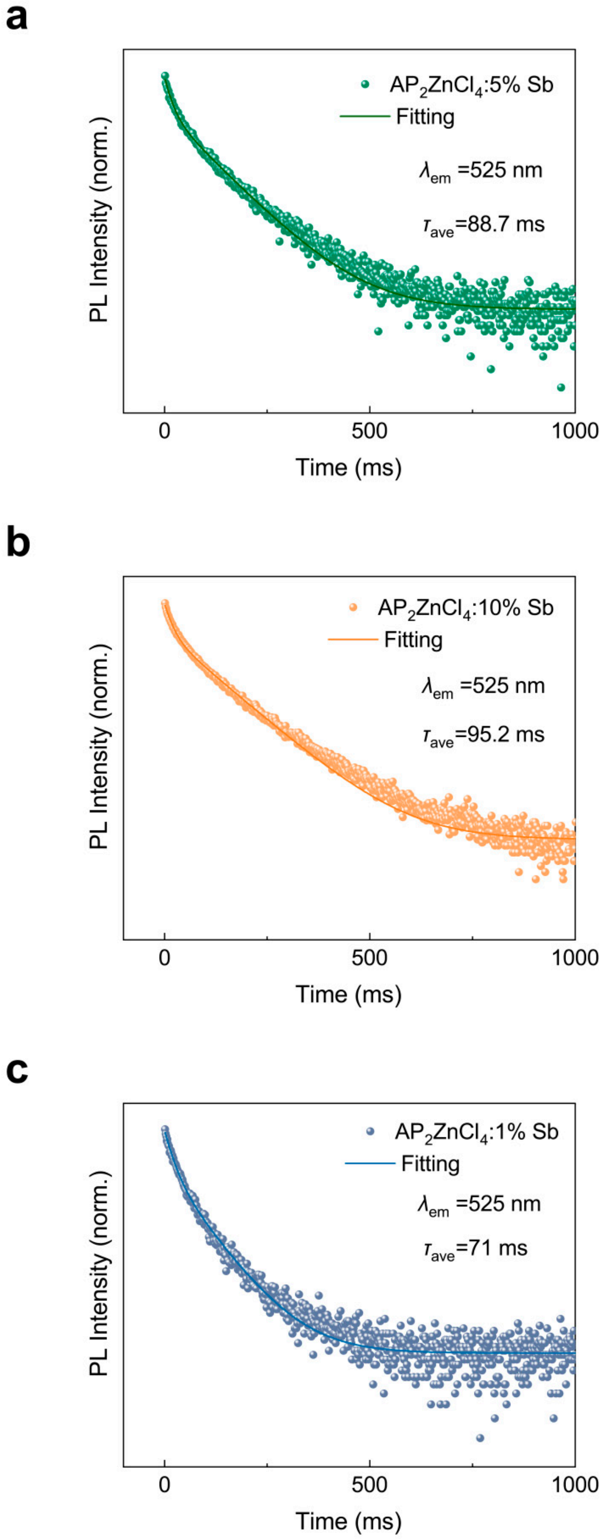

Figure S9. Time-resolved phosphorescence decay profiles of $(AP)_2ZnCl_4$:x%Sb monitored at λem = 525 nm. (a–c) Decay curves for the 5%, 10%, and 1% Sb samples, respectively, with average lifetimes of 88.7, 95.2, and 71 ms. Symbols denote the measured data and solid curves are the fits.

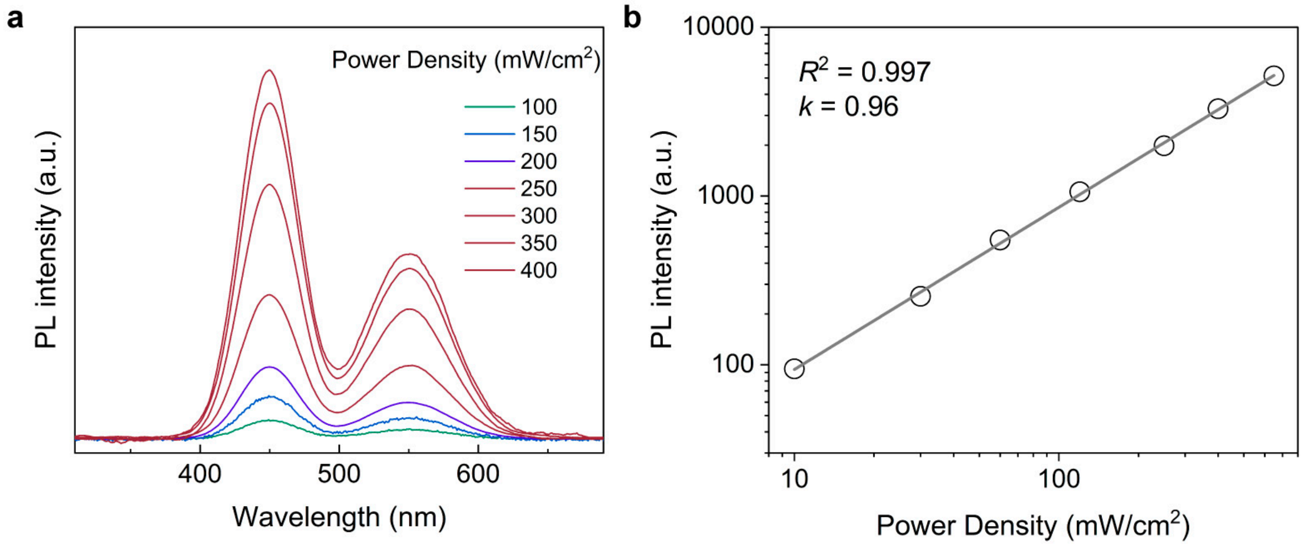

Figure S10. Power-dependent prompt PL spectra of the Sb-doped sample and the corresponding double-logarithmic fit of the integrated Sb-related long-wavelength emission. The fitted slope is k = 0.96 (R^2 = 0.997).

Image-based semi-quantitative analysis of time-gated readout

To provide a semi-quantitative validation of the composition-dependent temporal readout, the afterglow photographs of the 0%, 5%, and 15% nominal Sb compositions were analyzed using a consistent region-of-interest (ROI) procedure. All photographs used for this analysis were recorded under identical camera settings and acquisition conditions. A circular ROI of identical size was placed at the center of each sample, and the surrounding dark region was used for background correction. The background-corrected RGB signal was normalized to the corresponding value under continuous 365 nm excitation according to $I_{norm}(t) = \frac{I_{ROI}(t) - I_{bg}(t)}{I_{ROI}(UV\ on) - I_{bg}(UV\ on)}$. At 0.5 s, the normalized intensities of the 0%, 5%, and 15% Sb samples were 0.2927, 0.5297, and 0.1494, respectively. At 1.0 s, the corresponding values were 0.1186, 0.3652, and 0.0005, while at 1.5 s only the 5% Sb sample retained a measurable normalized signal (0.1677). These results provide semi-quantitative support for the composition-dependent persistence contrast used in the proof-of-concept time-gated decoding scheme. This image-based analysis is intended as representative support and does not replace repeated photometric calibration, cycling tests, or full statistical evaluation of decoding reliability.

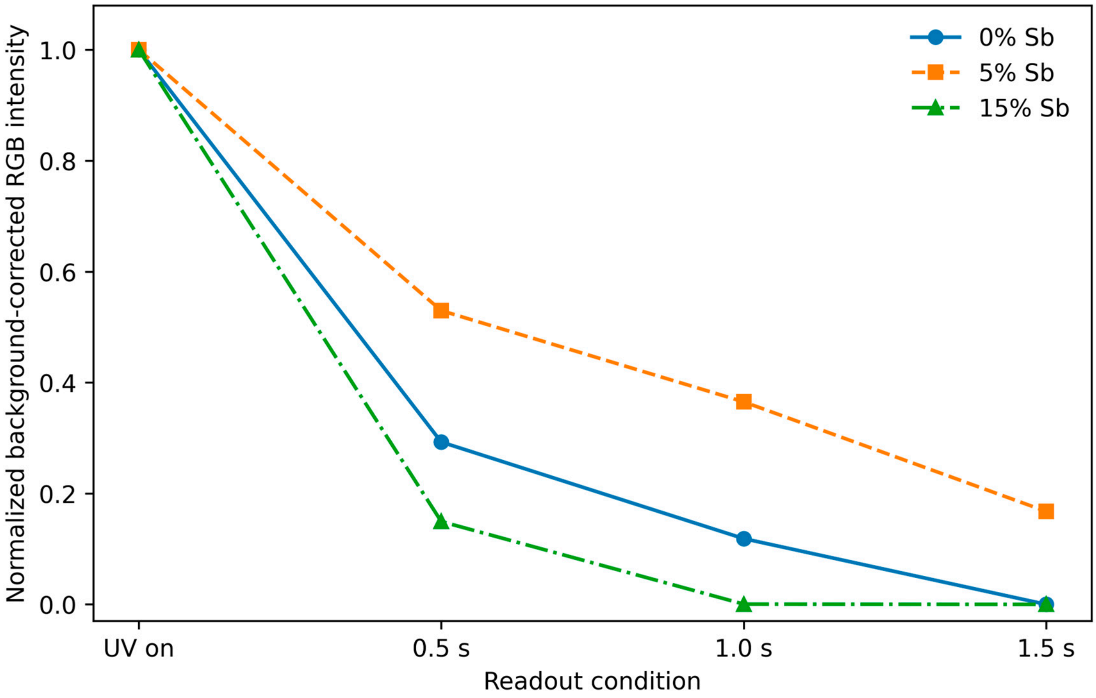

Figure S11. Semi-quantitative ROI analysis of the composition-dependent afterglow persistence used for time-gated optical readout. Background-corrected RGB intensities of the nominal 0%, 5%, and 15% Sb samples were extracted from photographs acquired under identical imaging conditions and normalized to the corresponding intensity under continuous 365 nm excitation. The analysis shows the fastest decay for the 15% Sb sample, intermediate persistence for the pristine sample, and the longest persistence for the 5% Sb sample, providing representative support for the temporal contrast used in Figure 8.

Experimental repeatability of delayed photoluminescence spectra

To further demonstrate experimental repeatability, three unnormalized delayed photoluminescence spectra of pristine $(AP)_2ZnCl_4$ were independently acquired under 360 nm excitation with matched optical settings. Two spectra were extracted from independently recorded excitation–emission matrices, and the third was obtained from an independent emission scan. The emission maxima are 505, 504, and 504 nm, giving an average peak position of 504.33 ± 0.58 nm. The coefficient of variation of the integrated intensity over 420–580 nm is 4.46%, and all pairwise Pearson correlation coefficients exceed 0.999. The close agreement in band shape, peak position, and integrated intensity confirms good repeatability under the present measurement conditions.

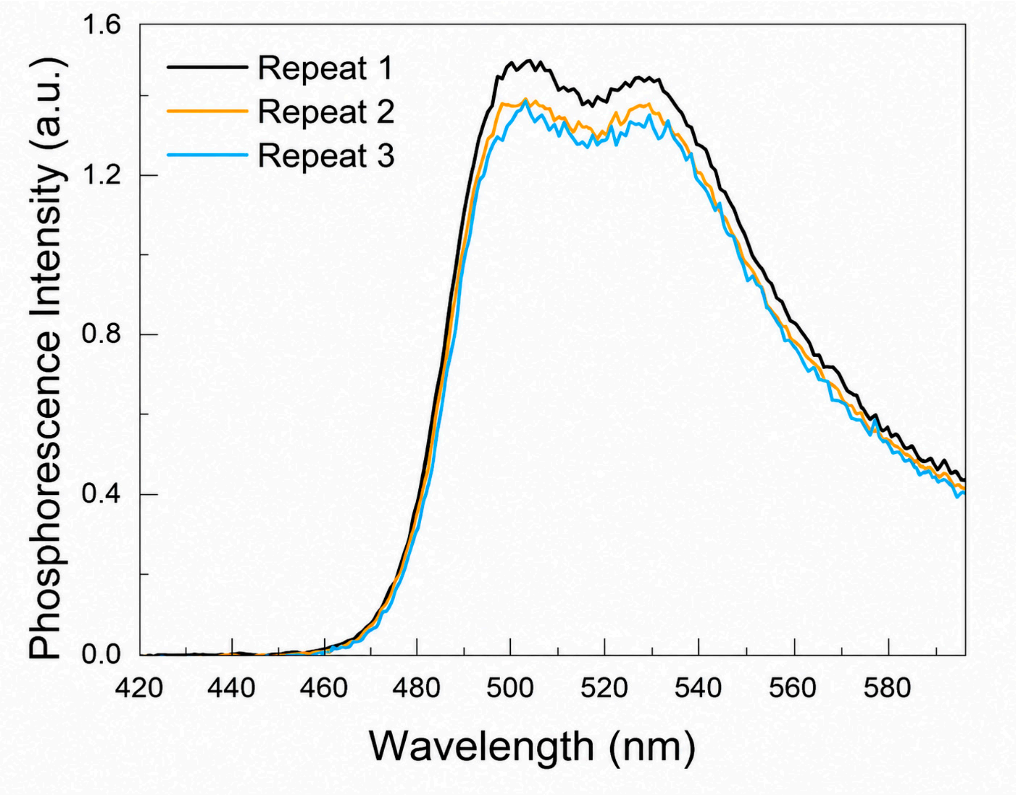

Figure S12. Experimental repeatability of the delayed photoluminescence spectra of pristine $(AP)_2ZnCl_4$ under 360 nm excitation. Three independently acquired, unnormalized spectra are shown. Two spectra were extracted from independently recorded excitation–emission matrices, and the third was obtained from an independent emission scan.
